# Developmental Disruption of *Erbb4* in *Pet1*+ Neurons Impairs Serotonergic Sub-System Connectivity and Memory Formation

**DOI:** 10.3389/fcell.2021.770458

**Published:** 2021-12-10

**Authors:** Candela Barettino, Álvaro Ballesteros-Gonzalez, Andrés Aylón, Xavier Soler-Sanchis, Leticia Ortí, Selene Díaz, Isabel Reillo, Francisco García-García, Francisco José Iborra, Cary Lai, Nathalie Dehorter, Xavier Leinekugel, Nuria Flames, Isabel Del Pino

**Affiliations:** ^1^ Neural Plasticity Laboratory, Príncipe Felipe Research Center, Valencia, Spain; ^2^ Developmental Neurobiology Unit, Instituto de Biomedicina de Valencia, IBV-CSIC, Valencia, Spain; ^3^ Bioinformatics and Biostatistics Unit, Príncipe Felipe Research Center (CIPF), Valencia, Spain; ^4^ Department of Psychological and Brain Sciences, Indiana University, Bloomington, IN, United States; ^5^ INMED, INSERM, Aix Marseille University, Marseille, France; ^6^ Institut de Neurobiology de la Méditerranée (INMED, UMR1249), INSERM, Marseille, France

**Keywords:** serotonin, ErbB4, NRG, memory, neuromodulation, neurodevelopmental disorders

## Abstract

The serotonergic system of mammals innervates virtually all the central nervous system and regulates a broad spectrum of behavioral and physiological functions. In mammals, serotonergic neurons located in the rostral raphe nuclei encompass diverse sub-systems characterized by specific circuitry and functional features. Substantial evidence suggest that functional diversity of serotonergic circuits has a molecular and connectivity basis. However, the landscape of intrinsic developmental mechanisms guiding the formation of serotonergic sub-systems is unclear. Here, we employed developmental disruption of gene expression specific to serotonergic subsets to probe the contribution of the tyrosine kinase receptor ErbB4 to serotonergic circuit formation and function. Through an *in vivo* loss-of-function approach, we found that ErbB4 expression occurring in a subset of serotonergic neurons, is necessary for axonal arborization of defined long-range projections to the forebrain but is dispensable for the innervation of other targets of the serotonergic system. We also found that *Erbb4*-deletion does not change the global excitability or the number of neurons with serotonin content in the dorsal raphe nuclei. In addition, ErbB4-deficiency in serotonergic neurons leads to specific behavioral deficits in memory processing that involve aversive or social components. Altogether, our work unveils a developmental mechanism intrinsically acting through ErbB4 in subsets of serotonergic neurons to orchestrate a precise long-range circuit and ultimately involved in the formation of emotional and social memories.

## 1 Introduction

Serotonin (also known as 5-hydroxytryptamine (5HT)) is a phylogenetically conserved signaling molecule (Hay-Schmidt, 2000) regulating diverse emotional, cognitive and neurovegetative functions. In mammals, serotonergic neurons distributed in the raphe nuclei of the brainstem are clustered in different groups topographically classified as B1–B9 (Dahlstrom and Fuxe, 1964). The largest group of serotonergic neurons allocate within the dorsal (B7 and B6) and median raphe (B8) nuclei (DRN and MRN, respectively) and develop exuberant axonal projections targeting almost every brain region.

A functionally diverse competence of DRN and MRN serotonergic circuits is thought to result from the development of extensive axonal projections reaching multiple brain targets. DRN and MRN serotonergic circuit formation is guided by intrinsic and extrinsic factors. For instance, general transcriptional mechanisms involved in serotonergic fate specification, i.e. *Lmx1b*, act at subsequent developmental stages to promote axon outgrowth from most serotonergic neurons (Donovan et al., 2019). Additional findings suggest that cell adhesion molecules (Cadherin 13) molecules and guidance cues (Eph5/ephrinA5) are involved in the negative regulation of axon outgrowth and/or pathfinding in addition to neuron proliferation of serotonergic neurons (Forero et al., 2017; Teng et al., 2017). In particular, high levels of ephrinA5 receptor expression in DRN has been described to repel axon growth in the hypothalamus and regulate proper arborization of serotonergic fibers in the olfactory bulb through repulsion by ephrinA5 (Teng et al., 2017).

Recent findings from multiscale analysis support the idea of a molecular, circuit and functional heterogeneity even within the DRN and MRN neurons. Electrophysiological, neuroanatomical and transcriptomic profiling suggest that DRN and MRN serotonergic neurons are highly heterogeneous (Okaty et al., 2015; Ren et al., 2019; Okaty et al., 2020; Senft et al., 2021). For example, neurons within the DRN have been described to segregate between circuits expressing the vesicular glutamate transporter 3 (Vglut3) preferentially innervating cortical areas and circuits expressing thyrotropin-releasing hormone (Trh) preferentially innervating subcortical nuclei (Ren et al., 2019) and regulating different behavioral demands (Ren et al., 2018). Additional studies showed restricted expression of the neuropeptide galanin in serotonergic neurons innervating the medial prefrontal cortex (Fernandez et al., 2016). However, although these findings were fundamental to parse out molecular profiles and assign them to projection-specificity, serotonergic neurons expressing Vglut3, Trh and/or galanin do not account for all DRN serotonergic circuits projecting to the forebrain. Thus, the molecular profiles of projection-defined serotonergic sub-systems targeting specific forebrain regions such as the thalamic sub-areas remain unresolved (Ren et al., 2019). Moreover, specific molecular mechanisms underlying sub-circuit development within the DRN or MRN are largely unknown.

NRG/ErbB4 signaling pathway has been thoroughly studied for its influence on the development of cortical GABAergic (Flames et al., 2004; Fazzari et al., 2010; Ting et al., 2011; Del Pino et al., 2013; Batista-Brito et al., 2017; Del Pino et al., 2017) and thalamocortical circuits (Lopez-Bendito et al., 2006) as well as in dopaminergic circuit function (Skirzewski et al., 2018). Genetic targeting previously revealed that ErbB4 is also expressed in subsets of serotonergic neurons (Bean et al., 2014). Nevertheless, whether ErbB4 is an intrinsic factor contributing to projection-defined serotonergic circuit development is unclear. Therefore, to gain a better understanding of the mechanisms underlying serotonergic circuit organization, we interrogate the functional relevance of *Erbb4*-expression for serotonergic circuit development and function. Here, we show that ErbB4 is co-expressed in subsets of adult 5HT neurons expressing the transcription factor *Pet1* required for serotonergic fate. We further demonstrate that ErbB4 is required for the axonal arborization of serotonergic projections in specific postsynaptic targets while dispensable for 5HT expression and global excitability in the DRN. Finally, we revealed that developmental deficiency of ErbB4 in serotonergic circuits impairs cognitive function related to specific types of memory in adult mice without affecting other emotional behaviors such as anxiety or coping behavior. Altogether, our findings link, for the first time, the NRG/ErbB4 signaling pathway with the organization of serotonergic sub-systems and unveils the specific role of this link in memory formation.

## 2 Materials and Methods

### 2.1 Animals

*Erbb4* ^*f/f;Pet1-Cre;Ai9f/*+^ were generated by breeding *Pet1-Cre* (Fev-Cre) lines (JAX stock #012712; RRID:MGI:3696982) (Scott et al., 2005) with mice carrying the loxP-flanked (f) *Erbb4* alleles (Golub et al., 2004) and *Ai9/tdTomato* lines (JAX stock #007909; RRID:MGI:J:155793) (Madisen et al., 2010). Control mice included mice carrying *Pet1-Cre* or *Erbb4^f/f^* alleles. All animal procedures were approved by the Ethics Committee (CIPF, Spain) and complied with the Spanish and European regulations for the use of laboratory animals.

### 2.2 Immunohistochemistry and Imaging Analysis

Mice (postnatal day P60–90) were transcardially perfused with 4% PFA, post-fixed for 2 h and sectioned with a freezing microtome. Immunohistochemistry for 5HT, ErbB4 and tdTomato was performed in 40 µm-thick sections using the following primary antibodies: rabbit anti-ErbB4 (0618 produced by Cary Lai), goat anti-5HT (Abcam 66047 (1:1,000), rabbit anti-5HT (1:5,000, Sigma #S5545, RRID:AB_477522) and rabbit anti-RFP (1:2,000, Rockland Cat# 600-401-379, RRID:AB_2209751). Secondary antibodies and Alexa555-conjugated streptavidin were purchased from Molecular Probes. Subsequently, sections were counterstained with DAPI and mounted with Mowiol. For the reconstruction of biocytin-filled neurons during *ex vivo* electrophysiology, 300 µm-thick brain sections were fixed overnight in 4% PFA at 4°C and incubated with Alexa488-conjugated streptavidin (1:500, Thermo Fisher Scientific Cat# S11223, RRID:AB_2336881).

Imaging of fluorescently labelled axonal arbors in target regions of the serotonergic system was performed with 20X 0.80NA objective in an Aperio Versa slide scanner (Leica Biosystems) or with the DMI-4 SP8 confocal microscope (Leica Biosystems). Following the neuroanatomical features (Franklin and Paxinos, 2008) in DAPI counterstained brain sections, images of 1,500 × 1,500 pixels (for the lateral hypothalamus, periventricular nucleus of the thalamus or corpus callosum) or manually delimited brain regions (for the dentate gyrus, CA3 and CA1) were extracted from two single z-planes per area and from 3 areas per mouse in ImageScope (Leica Biosystems). The percentage of area occupied by fluorescent signal was performed in Fiji/ImageJ (RRID:SCR_002285) applying the same threshold over the background for all genotypes. For analysis of relative density of fluorescently labeled fibers, a threshold of 30 and 50 arbitrary units of intensity over the background was applied in images acquired with AperioVersa and with the confocal microscope, respectively. Background signal was quantified from 3 different regions of the field of view without tdTomato+ neuropil labelling. For cell counting, colocalization analysis and morphological reconstruction of biocytin-filled neurons, images were acquired with a 20X 0.75NA objective in a DMI-4 SP8 confocal microscope (Leica Biosystems). Colocalization was quantified from two different optical planes per section. For analysis of fluorescence intensity, images were acquired at 16bit depth. For neural tracing and morphological reconstruction, stacks were acquired at 1 µm step size and the SNT 3.1.109 tool of Fiji/ImageJ was employed (Arshadi et al., 2021).

### *2.3 Ex Vivo* Electrophysiology

*Erbb4* ^*+/+;Pet1-Cre;Ai9f/*+^ and *Erbb4* ^*f/f;Pet1-Cre;Ai9f/*+^ male mice (12–13 weeks-old) were used. First, mice were anaesthetized with isoflurane and perfused with ice-cold artificial cerebrospinal fluid (aCSF) medium containing (in mM): 87 NaCl, 25 NaHCO_3_, 5 D-(+)-glucose, 65 sucrose, 2.5 KCl, 1.25 NaH_2_PO4, 0.5 CaCl_2_, 7 MgCl_2_, 5 ascorbic acid and 3.1 pyruvic acid saturated with 95% CO_2_ and 5% O_2_(pH 7.4). Then, mice were decapitated and brain was removed and placed in oxygenated aCSF. 300-µm coronal slices were obtained with a vibratome (Microm HM 650 V) and transferred to a chamber with aCSF solution consisting of: 125 NaCl, 25 NaHCO_3_, 25 D-(+)-glucose, 2.5 KCl, 1.25 NaH_2_PO_4_, 2 CaCl_2_, 1 MgCl_2_, 1 ascorbic acid and 4 pyruvic acid saturated with 95% CO_2_ and 5% O_2_ (pH 7.4). Slices were allowed to recover at 35°C for 30 min. Patch clamp recordings in whole-cell configuration were performed at 25°C using a potassium gluconate-based intracellular solution containing (in mM): 135 K-gluconate, 10 HEPES, 10 Na-phosphocreatinine, 4 KCl, 4 MgATP, 0.3 NaGTP and adjusted to 290mosmol/l and pH7.2–7.4. Biocytin was added to the internal solution at a concentration of 1.5–2.5 mg/ml for *post hoc* immunohistochemistry. tdTomato expressing neurons were visualized with an upright microscope (Olympus BX51WI; RRID:SCR_018949) equipped with an ORCA-ER CCD Camera (Hamamatsu), a 40X/0.8 nA water-immersion objective (Olympus), a X-Cite 120Q fluorescence lamp and infrared-differential interference optics contrast for bright field imaging. Micro-pipettes of 10–12 MΩ were pulled from borosilicate glass using a vertical P-10 puller (Narishige). Data was acquired and sampled at 20 kHz using a MultiClamp 700B (Molecular Devices, RRID:SCR_018455), a digitizer Digidata 1440A (Molecular Devices, RRID:SCR_021038) and pClamp software (Molecular Devices, RRID:SCR_011323).

Intrinsic electrophysiological properties were calculated using different current-clamp protocols. Resting Membrane Potential (V_rest_) was measured after breaking into the cell. Input Resistance (R_in_) was obtained using 500 ms hyperpolarizing current steps of ∆5 pA from −20 to 0 pA. Rheobase, action potential kinetics and input-output function were measured by applying 500 ms depolarizing current steps of ∆ 10 pA from 0 to +180 pA in neurons held at −70 mV. EasyElectrophysiology software (EasyElectrophysiology Ltd., RRID:SCR_021190) (Garcia et al., 2014) was used for the analysis of electrophysiological properties. Threshold potential was defined as dV/dt = 10 mV/ms using first derivative method. AP threshold was defined in a region of 10 ms before peak. Rise and decay time were determined in a minimum-maximum cutoff percentage of 10–90%. Half-width is calculated as the time between rise and decay at half amplitude. fAHP was detected in a search region of 0–9 ms after peak and mAHP in a region of 30–70 ms. Both values were calculated as baseline minus the minimum value within each region. Rheobase was defined as the minimum current injected to obtain the first AP from neurons held at −70 mV. Maximum firing frequency was obtained from spike frequency discharge upon +190 pA current injected. Inter-spike interval (ISI) was calculated as the difference between first and second AP at first step with ≥2AP. Spike frequency adaptation was obtained by dividing the first ISI by the final ISI of the trace with ≥4AP. Input-output curve was plotted averaging the number of AP detected for each neuron at each current step.

### 2.4 Mouse Behavioral Analysis

Adult *Erbb4* ^*f/f*^ and *Erbb4* ^*f/f;Pet1-Cre*^ male and female littermates (P60) were used for behavioral tests. Mice were maintained under standard housing conditions in 12 h dark/light cycles with food and water *ad libitum*. All different tests were implemented during the light phase blind to the genotype. Mice were handled and habituated to the experimenter for 3 days before the behavioral assays. All behavioral tests were separated at least by 24 h. The order of tests was as follows: open field, habituation to open field, Y-maze, sociability test, elevated plus maze (EPM), dark-light box and nest building test. Contextual fear conditioning, prepulse inhibition of the startle response (PPI), forced swimming and tail suspension test were performed in separated batches of animals. Testing apparatuses were cleaned with a solution of 70% ethanol in water after each trial to avoid olfactory cues. Behavioral tasks were recorded with a Logitech C270 webcam HD camera and analysed using Ethovision® XT software (Noldus, RRID:SCR_000441) unless otherwise stated.

#### 2.4.1 Open Field

Spontaneous locomotor activity was measured in an open field apparatus. It consisted of a rectangular chamber of 48 × 48 × 48 cm that was made of plastic under uniform light conditions (70 lux). Mice were allowed to explore the arena for 10 min. The arena was delimited for analysis into two different regions: centre (square area of 30 × 30 cm equidistant from the walls) and the remaining borders. The time spent, velocity, distance as well as transitions between the zones were calculated with Ethovision® XT. Habituation to the open field was performed 1 day after first exposure to the arena in the same conditions as previously described.

#### 2.4.2 Spontaneous Alternation Task in the Y-Maze

Spontaneous alternation task was performed in a transparent Y-maze (50 × 8 cm each arm). Mice were placed into the centre of the maze and allowed to freely explore for 8 min. The exploration was recorded and the sequence of mouse entries in each arm was analysed *post hoc*. The spontaneous alternation behavior was calculated as the number of triads containing entries into all three arms divided by the maximum possible alternations as follows:

$$I_{alternation}=\frac{total\;alternations}{\left(total\;entries\;-2\right)}\times 100$$

#### 2.4.3 Sociability and Preference for Social Novelty Test

Sociability and preference for social novelty was assessed in a three chambered arena (50 × 25 × 25 cm) with one cylinder in each side chamber. The cylinders (wire cup-like containers) allowed auditory, visual and olfactory interaction between mice. This paradigm consisted of three phases: habituation, sociability phase (S1) and social novelty (S2). During habituation, mice are allowed to explore the arena with the cylinders empty for 5 min. We routinely control that animals do not have a preference for any context by measuring the time spent in the lateral chambers during the habituation phase. In the S1 phase mice are allowed to explore the arena for 10 min with an unfamiliar male mouse and an object randomly allocated inside each side chamber´s cylinder. In the S2 phase, a new non-familiar mouse is placed instead of the object and the test mouse is allowed to explore both subjects for additional 10 min. The chamber in which the S1 or S2 mice were placed was counterbalanced between trials, to avoid spontaneous preference.

To assess time sniffing the social and non-social cylinders or familiar versus novel subject, a circular area of 15 cm diameter encompassing each cylinder was set in Ethovision® XT and exploration time was defined as the time during which the mouse nose is inside the area (sniffing zone). A preference index (I_p_) was calculated for each phase by subtracting the time sniffing the non-social area from the time sniffing the social area. For S2 the I_p_ was calculated by subtracting the time sniffing the area of the unfamiliar subject from the time sniffing the area with the familiar subject.

$$I_{p}=\frac{t_{unfamiliar\;area}\;-t_{familiar\;area}}{t_{unfamiliar\;area}+t_{familiar\;area}}$$

A positive score in the preference index indicates a preference for the social stimuli in S1 phase or for the novel (unfamiliar) subject in the S2 phase, and a negative score indicates a preference for the non-social stimuli in the S1 phase or a deficit in social memory in the S2 phase.

#### 2.4.4 Elevated Plus Maze

The elevated plus maze consisted of four arms (50 × 10 cm) elevated 50 cm above the floor. The plus maze had two closed arms with black acrylic glass walls (30 cm high) and two open (wall-free) arms connected by a central platform. Indirect illumination provided 100 lux to the open arms and 15 lux to the closed arms. Mice were gently placed in the centre of the maze and their behavior was recorded for 5 min. Time spent and the number of entries in each arm was quantified *post hoc* with Ethovision® XT.

#### 2.4.5 Dark-Light Box

This test is based on the innate aversion of rodents to avoid open and strong illuminated zones. The test apparatus consisted of two boxes (25 × 25 cm each) connected by a small aperture: the light box, (open box with direct illumination of 450 lux) and the dark box (opaque). Mice were placed facing the dark box and their behavior was recorded for 5 min. Mice were video-tracked in the light box post hoc with Ethovision®XT software. Time spent in the light box as well as the number of transitions between the boxes were quantified, and a ratio of time in dark box over time in light box was calculated. A transition is considered when the head of the mouse enters the other zone.

#### 2.4.6 Prepulse Inhibition Test

Response and inhibition of animals after non-startling pulse (prepulse) were recorded in a restrictive acoustic startle response system (The StartFear Combined System; Panlab, Spain). All mice were habituated to the testing chamber without background noise 2 days before the PPI test. For each test session, mice were placed in the cage for 5 min in order to acclimatize to a background noise of 70 dB. After that, three 20 ms pulse stimuli were delivered at 120 dB to define the basal level of the startle reaction for each mouse. Then, mice received blocks of 4 different trials presented pseudo-randomly. Each of them consisted on four trial types: a first acoustic pulse of 120 dB followed of three different 20 ms prepulse trials of 80, 85, and 90 dB each. The maximum startle amplitude of response to acoustic pulses for each mouse were used for the calculation of the percentage of PPI, divided by the average amplitude of startle response for each different trial, as follows:

$$\%\;PPI\;=\frac{\left(startle\;amplitude\;in\;the\;startle\;trial-startle\;amplitude\;in\;the\;prepulse\;trial\right)}{startle\;amplitude\;in\;the\;startle\;trial}\times 100$$

#### 2.4.7 Nesting Behavior

To study the ability of mice to build the nest, two pieces of nesting material made of cotton fibber (3 × 1 cm each) were introduced in the cage in which the mouse was individually housed. Nest quality was imaged at 1, 2, and 3 h after placement of the nesting material. The unused pieces of nesting material were weighted at each timepoint (t). The percentage of unused cotton was calculated to measure organizational behavior at different timepoints as follows:

$$\text{\% Unshredded cotton }=\frac{weight_{nesting\;material\;at\;t\left(x\right)}}{weight_{nesting\;material\;at\;t\left(0\right)}}\times 100$$

#### 2.4.8 Contextual Fear Conditioning

Fear memory was tested using the Fear Conditioning system (Maze Engineers, Boston, USA) in an acrylic plastic cage of 17 × 17 × 25 cm placed over a shock generator grid. During contextual fear conditioning test (CFC), mice were first allowed to explore the context (conditioned-stimulus) consisting of the transparent chamber, distal visual cues and olfactory cues (ethanol 70%) during 3 min. This was followed by three 0.7 mA foot-shocks (unconditioned stimulus, US) of 1 s given with a 30 s inter-stimulus interval. Memory retrieval was tested 1 day after conditioning, placing mice in the same context without the US during 5 min. Memory performance was defined as the percentage of immobility over total time. Immobility was manually quantified and quantifications were validated with Ethovision® XT.

#### 2.4.9 Forced Swimming Test

Mice were placed in a transparent cylindrical tank (12 cm of diameter) made of glass filled with water at room temperature. The height of the tank was 23.5 cm to prevent the mice from scaping. The tank was illuminated with 56 lux. Then, mice were gently and slowly placed in the water and their escape related mobility was recorded with a video camera for 6 min. Percentage of immobility over the total time was hand-measured by two different researchers blind to genotype.

#### 2.4.10 Tail Suspension Test

Tail suspension test was carried out to assess depression-like behavior. Mice were suspended by their tails with tape in a position that prevented them from escaping. Escape-oriented behavior was recorded during 6 min. Percentage of immobility over the total time was hand-measured by two different researchers blind to genotype.

### 2.5 Analysis of Published Single-Cell RNAsequencing Data and Visualization

Dataset were downloaded from NCBI database (GSE135132) from Ren et al. (2019) article. RStudio (R version 4.0.2, RRID:SCR_001905) software was used for assessing scRNAseq analysis. *Seurat* package (version 4.0.1, RRID:SCR_016341) was used for performing filtering, highly-variable gene-selection, dimensionality reduction and clustering. Cells with fewer than 300 detected genes and genes detected in fewer than 3 cells were removed from original datasets for following analysis. *SCTransform* function was used for normalization, scale and detection of variable genes. Counts were log-normalized using the natural logarithm of 1 + counts per million [ln (CPM+1)]. PCA, tSNE and UMAP dimensional reductions were performed considering significant dimensions. Clusters were defined based on shared nearest neighbor (SNN) using *FindClusters* function considering PCA dimensional reduction. *Pet1 (Fev)* and *Erbb4* transcripts in the datasets were visualized using *FeaturePlot* function of tSNE reduction. The percentage of *Pet1* cells expressing *Erbb4* was determined used custom function. *Erbb4* transcripts in *Pet1* cells was plotted using *VlnPlot* function.

### 2.6 Statistics

Statistical analyses were carried out with the GraphPad Prism 9 software (RRID:SCR_002798) and with R programming. The study of the coordinates (Euclidean distance and nearest neighbor distance) and principal component analysis were performed with R. All data are presented as mean ± SEM. Biological replicas (N values) are different animals (behavior and immunohistochemistry) or cells from >3 different brains (electrophysiology) derived from >3 different litters. Statistical methods were used to predetermine the sample size. Randomization was not used. Experiments and analyses were performed blind to genotype by two different experimentalists. Differences were considered significant when *p* < 0.05 (*), *p* < 0.01 (**) or *p* < 0.001 (***). The data were analyzed with parametric tests (Student’s t test or ANOVA), when the datasets fulfilled the assumptions of normality (Kolmogorov-Smirnov test) and homoscedasticity (Levene’s test). Non-parametric tests for independent groups or Mann-Whitney test were applied when normality was not reached and Kolmogorov-Smirnov test was used when comparing probability distributions. Statistical analysis of the majority of experiments included the sex perspective except when appropriately indicated. For this purpose: animals of both sexes were included in the samples of each experiment. Pooled analysis for both sexes was carried out after performing stratified analysis by sex and validating that the patterns of data distribution were common in males and females.

## 3 Results

### 3.1 ErbB4 Is Expressed in Molecularly Defined Subsets of +*Pet1*+ Neurons of the Dorsal Raphe Nuclei

**FIGURE 1:**
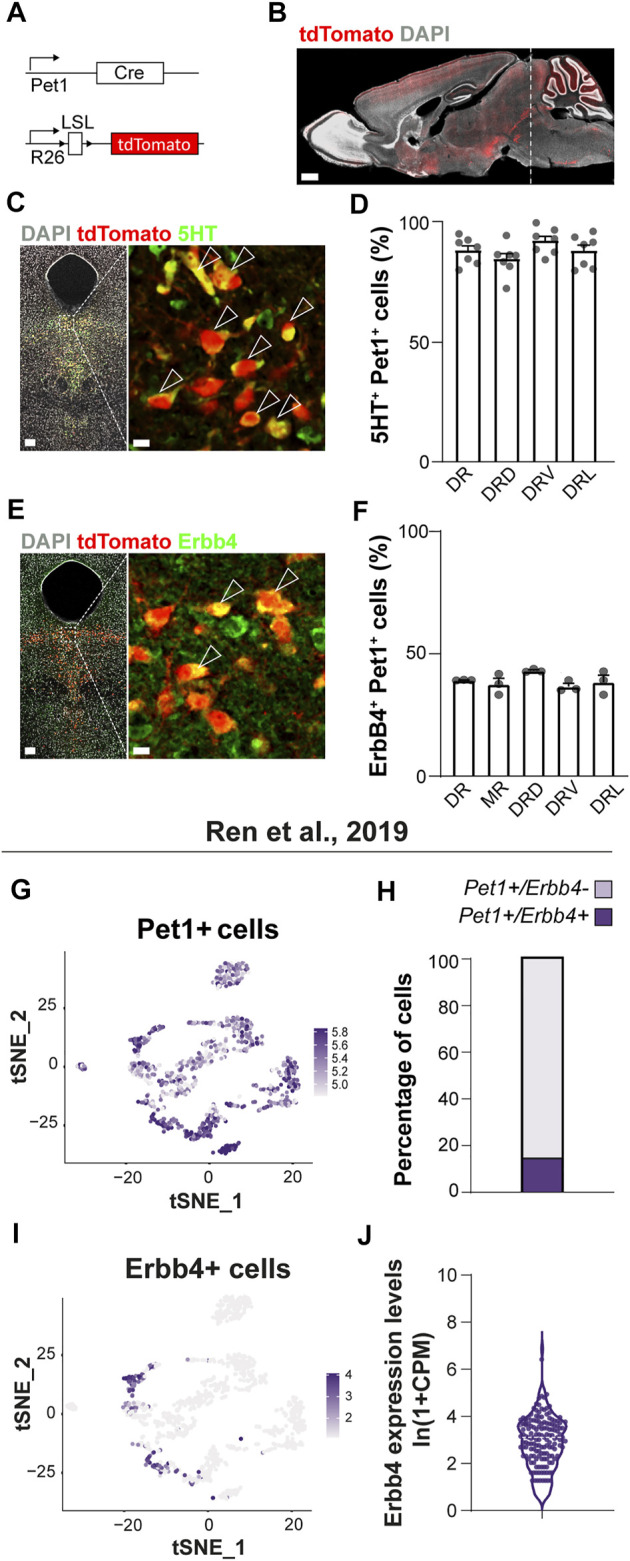
*Erbb4* expression in serotonergic neurons of the dorsal raphe labelled in *Pet1*-reporter mice. **(A)** Genetic strategy to generate *Pet1*-reporter mice. LSL: loxP-STOP-loxP. **(B)** Sagittal brain section of an adult *Pet1*-reporter mouse showing tdTomato-labelled projections and 5HT neurons located in the raphe nuclei (dashed line). **(C)** Confocal images of 5HT immunohistochemistry and *Pet1*-tdTomato+ neurons in the dorsal raphe nucleus (DR). **(D)** Quantification of percentage of colocalization of 5HT+ among *Pet1*-tdTomato+ cells in the DR and in each DR subregion (*n* = 7 brains from 4 different litters) **(E)** Confocal image of ErbB4 immunolabelling with *Pet1*-tdTomato+ neurons in the DR. **(F)** Quantification of percentage of colocalization of ErbB4+ cells among *Pet1*-tdTomato+ cells in the MR, DR and in each DR subregion (*n* = 3 brains from 3 different litters). **(G)** tSNE plot showing *Pet1* transcripts in the different clusters from the scRNA-seq dataset of Ren et al. (2019). Cells are colored according to log-normalized *Pet1* transcripts. Color legend reflects expression values of *Pet1* ln (CPM+1). **(H)** Percentage of cells *Pet1*+ from the Ren et al. (2019) study presenting *Erbb4* transcripts. (15.41% of total *Pet1*+ cells display *Erbb4* transcripts, i.e. 153 *Erbb4*+ cells from 999 *Pet1*+ cells). **(I)** tSNE plot showing *Erbb4* transcripts in the different clusters from the original dataset. Cells are colored according to log-normalized *Erbb4* transcripts. Color legend reflects expression values of *Erbb4* in ln (CPM+1). **(J)** Violin plot representing the cell distribution according to number of *Erbb4* transcripts (ln (CPM+1)) within *Pet1*+/ErbB4+ cells (average from 153 *Erbb4*+ cells: 2.82 ± 0.09 reads (mean ± SEM)). DRD: dorsal region or the DR, DRV: ventral region of the DR, DRL: lateral region of the DR. Scale bars in (**B**): 1000 µm and in **(C,E)**: 100 and 10 µm. Data are represented as mean ± SEM.

To understand whether ErbB4 is involved in serotonergic circuit development, we employed mouse genetics to label serotonergic neurons with a fluorescent protein tdTomato reporter controlled by a Cre recombinase driven by *Pet1* promoter (*tdTomato* ^*f/+;* *Pet1* *-*Cre mice^, hereafter named *Pet1*-reporter mice) (Figure 1A), a determinant of serotonergic fate (Hendricks et al., 1999). *Pet1*-reporter mice allow the specific genetic targeting of a major population of serotonergic neurons within the raphe nuclei (Figure 1B) as shown by colocalization analysis between tdTomato and 5HT in the dorsal raphe nucleus (DRN) (86 ± 2% 5HT+ among *Pet1*+tdTomato+ cells) (Figures 1C,D) (77 ± 3% of *Pet1*+tdTomato+ among 5HT+ cells) (data not shown).

We then evaluated whether serotonergic neurons from the *Pet1* lineage express ErbB4. *Erbb4:CreERT2* reporter mice suggest ErbB4 expression in approximately 20% of 5HT neurons in the DR (Bean et al., 2014; Zhang et al., 2020). To validate this finding *in situ* at protein level, we employed immunohistochemistry with a previously validated anti- ErbB4 antibody (Zhu et al., 1995; Del Pino et al., 2013) in *Pet1*-reporter mice (Figure 1E). Quantification of percentage of ErbB4-expressing neurons within the *Pet1*+tdTomato+ cells showed that a subset of *Pet1*+ cells colocalized with ErbB4 in the DRN (39%) and similar percentages of colocalization were observed in each of the DRN subregions (DRD: 43%; DRV: 37%; DRL: 38%) and in the MRN (B8) (37%) (Figure 1F). In addition, to elucidate whether *Pet1*+ErbB4+ are serotonergic, we performed immunohistochemistry for 5HT as well as ErbB4 onto *Pet1*-reporter mice (Supplementary Figure S1). Quantifications *Pet1*+/ErbB4+/5HT+ and *Pet1*+/ErbB4+/5HT- neurons revealed that 95 ± 2% and 85 ± 5% of *Pet1*+ErbB4+ cells are serotonergic in the DRN and MRN respectively (Supplemetary Figure S1). These data suggested that ErbB4 is present in the DRN/MRN and marks a subpopulation of *Pet1*+ serotonergic neurons. Notably, ErbB4 neurons that are *Pet1*-negative and express 5HT can be found intermingled with *Pet1*-positive neurons (data not shown), suggesting that *Pet1*+ErbB4+ cells represent a subset of ErbB4-expressing serotonergic population.

Next, we aimed to better characterize the molecular identity of adult ErbB4+ serotonergic neurons taking advantage of transcriptomic data from recent single cell RNAseq of adult Sert and/or *Pet1*-expressing serotonergic neurons (Okaty et al., 2020; Ren et al., 2019). From 999 to 2,350 sequenced cells expressing *Sert* and/or *Pet1* in the Ren et al., 2019 (Ren et al., 2019) and Okaty et al., 2020 studies, respectively, *Erbb4* transcripts where detected in 15 and 37% of all cells (Figure 1; Supplementary Figure S2). Importantly, cells displaying *Erbb4* transcripts are not randomly distributed in the tSNE representation but locate mostly in four out of 13 clusters from the Ren et al., 2019 dataset (Figures 1G–J) and eight out of 14 clusters from the Okaty et al., 2020 dataset (Supplementary Figure S2). In addition, a close inspection of the molecular identity of *Pet1*-expressing neurons clustered by scRNAseq of the Ren et al., 2019 study (Ren et al., 2019) (Supplementary Figure S3), suggests that ErbB4-expressing neurons belong to clusters mapping to both the DRN as well as MRN. Specifically, *Erbb4* transcripts are present in cells from the cluster expressing the Iruquois Homeobox 2 (*Irx2*) and Tachykinin3 receptor (*Tacr3*) which was mapped to MRN in the original study (Ren et al., 2019) (Supplementary Figure S3A). In agreement with this, we performed a colocalization analysis between *Pet1*+tomato+ and ErbB4 immunolabelled neurons and found that 22 ± 4% *Pet1*+ were ErbB4+ in the MRN (data not shown). These data strongly suggest that ErbB4 expression, despite being anatomically distributed throughout the different regions of the DRN and MRN, correspond to specific 5HT molecular subtypes of neurons.

### 3.2 *Erbb4* Deletion From *Pet1*+ Neurons Does Not Affect Serotonergic Neuron Migration

**FIGURE 2:**
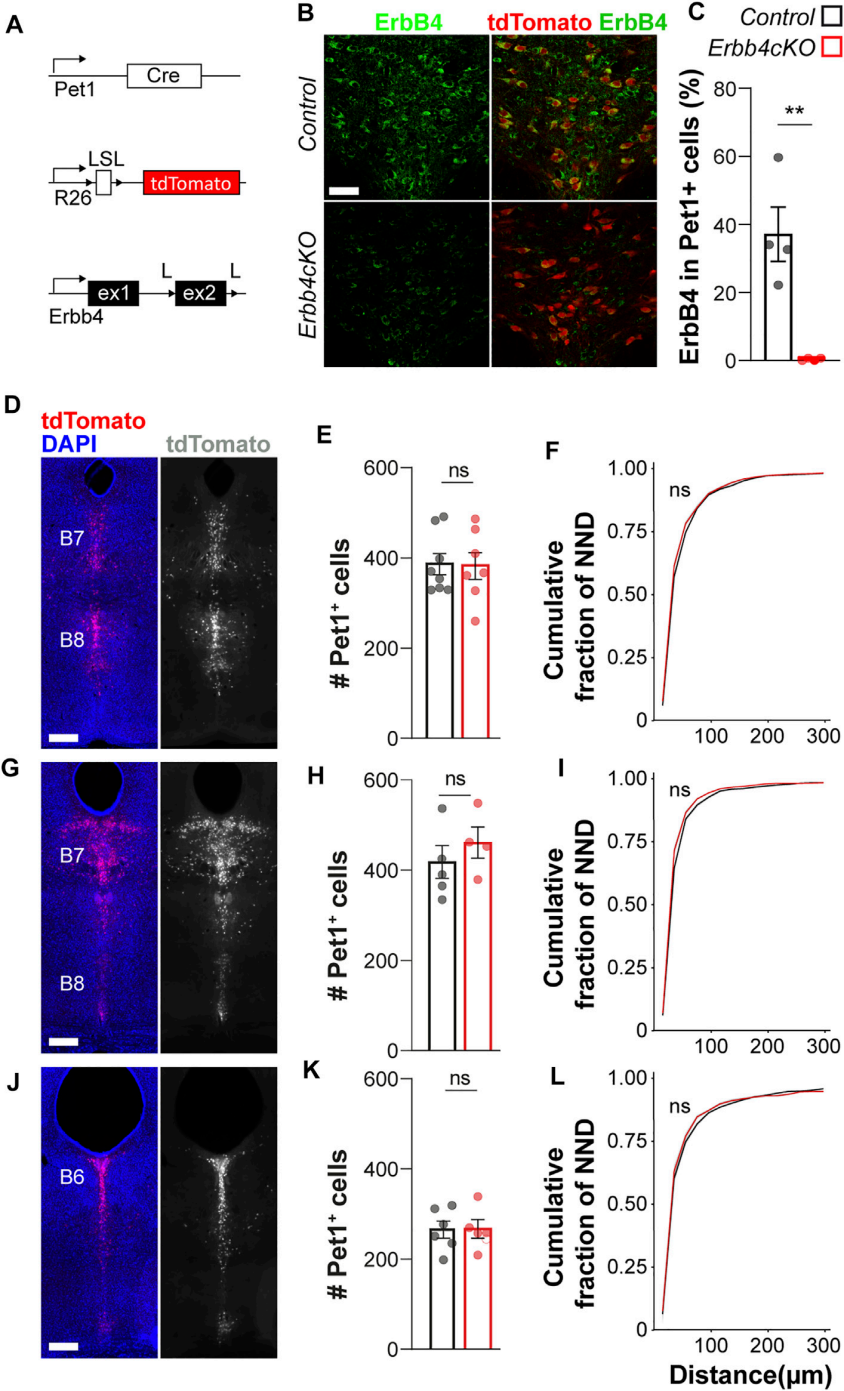
ErbB4 is not required for *Pet1*+ serotonergic neuronal migration. **(A)** Genetic strategy to generate *Erbb4cKO* mice. L: loxP, LSL: loxP-STOP-loxP site. **(B)** Confocal images of ErbB4-expressing and *Pet1*-driven tdTomato-expressing neurons in the dorsal raphe of control and *Erbb4cKO* mice. (*n* = 4 brains in control and *n* = 4 in *Erbb4cKO* mice from 3 different litters) **(C)** Quantification of percentage of colocalization of ErbB4+ and tdTomato+ neurons shows a significant reduction of colocalizing cells in *Erbb4cKO* mice when compared to control mice. **(D–L)** Distribution of *Pet1*-tdtomato+ neurons in the dorsal raphe nucleus (DRN). Coronal brain sections showing distribution of tdTomato+ neurons assessed at different levels of the DRN: Bregma −4.48 mm and **(D)** Bregma −4.72 mm **(G)** corresponding to B7/B8 nuclei as well as Bregma −5.02 mm **(J)** corresponding to B6. **(E, H, K)** Quantification of total tdTomato+ neurons found at each coordinate as depicted in **(D, G, J)** in the DRN of control and *Erbb4cKO* mice shows non-significant differences (*n* = 5–8 control and *n* = 4–7 mutant brains from 3 different litters). **(F, I, L)** Cumulative fraction of NNDs measured at each coordinate in the DRN shows non-significant differences in cell distribution (*n* = 5–8 control and *n* = 4–7 mutant brains from 3 different litters). Scale bars in **(B)**: 50 µm and in **(D, G, J)**: 500 µm. Data are represented as mean ± SEM. ns: not significant differences; **: *p* < 0.01. t-test or Kolmogorov-Smirnov test.

Having identified that ErbB4 is expressed in a subset of adult DRN *Pet1*+ neurons, we examined whether ErbB4 is involved in serotonergic circuit development. To address the contribution of ErbB4 to serotonergic system formation, we generated ErbB4^*f/f;*Pet1*-Cre;Ai9f/*+^ mice (hereafter named ErbB4cKO mice) in which the exon 2 of the *Erbb4* gene, is excised in serotonergic neurons since early fate specification using the *Pet1*-Cre-recombinase (Figure 2A). By performing immunohistochemistry against ErbB4 and quantifying the colocalization between *Pet1*+tdTomato+ cells and ErbB4 expressing cells, we observed a robust reduction in the percentage of *Pet1*+tdTomato+ neurons displaying high immunofluorescent signal for ErbB4 in the adult DRN in *Erbb4cKO* mice when compared to controls (*Erbb4* ^*+/+;*Pet1*-Cre;Ai9f/*+^ mice) (control: 37 ± 8%; *Erbb4cKO:* 1 ± 0.2% ErbB4+ among tdTomato+ cells; *p =* 0.003) (Figures 2B,C), showing that *in vivo* Erbb4-deficiency in serotonergic neurons can be efficiently achieved with this genetic strategy.

The ligands of ErbB4, Neuregulin 1 (*Nrg1*) and Neuregulin 3 (*Nrg3*), mediate long and short-range attraction of migrating GABAergic interneurons expressing ErbB4 in the forebrain (Zhang et al., 1997; Flames et al., 2004; Bartolini et al., 2017). Recent studies suggest that *Nrg3* is expressed in the roof plate early during morphogenesis E9–E11 (La Manno et al., 2021). At E9–E11, serotonergic neurons migrate from the ventricular zone close to the floor plate to the medial hindbrain (Hawthorne et al., 2010). Thus, we asked whether NRG/ErbB4 signaling would be involved in the migration and final cellular allocation of serotonergic neurons within the raphe nuclei. To tackle this question, we quantified the total number of *Pet1*-driven tdTomato+ cells in three different coordinates across the dorsal-caudal axis of the DRN corresponding to two different rostro-caudal levels of the B7 nucleus and the rostral B6 and MRN (B8). We observed that the total number of tdTomato+ neurons was not significantly different between control and *Erbb4cKO* mice at any of the neuroanatomical coordinates (*p >* 0.05, *t-test*) (Figures 2D,E,G,H,J,K; Supplementary Figure S4), indicating that migration and survival of serotonergic neurons is not affected by loss of ErbB4. In addition, cellular distribution of *Pet1*+ serotonergic neurons in the DRN and MRN was assessed by obtaining the spatial coordinates of each tdTomato+ cell within the DRN and MRN, respectively and calculating the distance from each tdTomato+ cells to its nearest neighbor (nearest neighbor distance or NND) at each coordinate. Spatial distributions were compared in cumulative fractions of NND for each population and did not reveal significant differences for any of the three neuroanatomical coordinates analyzed between control and *Erbb4cKO* mice (*p > 0.05, Kolmogorov-Smirnov test*) (Figures 2F,I,L) (Supplementary Figure S4). Altogether these data suggested that ErbB4 is dispensable for *Pet1*+ serotonergic neurons to migrate and allocate in the DRN and MRN (B8).

### 3.3 *Erbb4*-Loss in *Pet1*+ Neurons Impairs the Long-Range Connectivity of Serotonergic Sub-Systems

**FIGURE 3:**
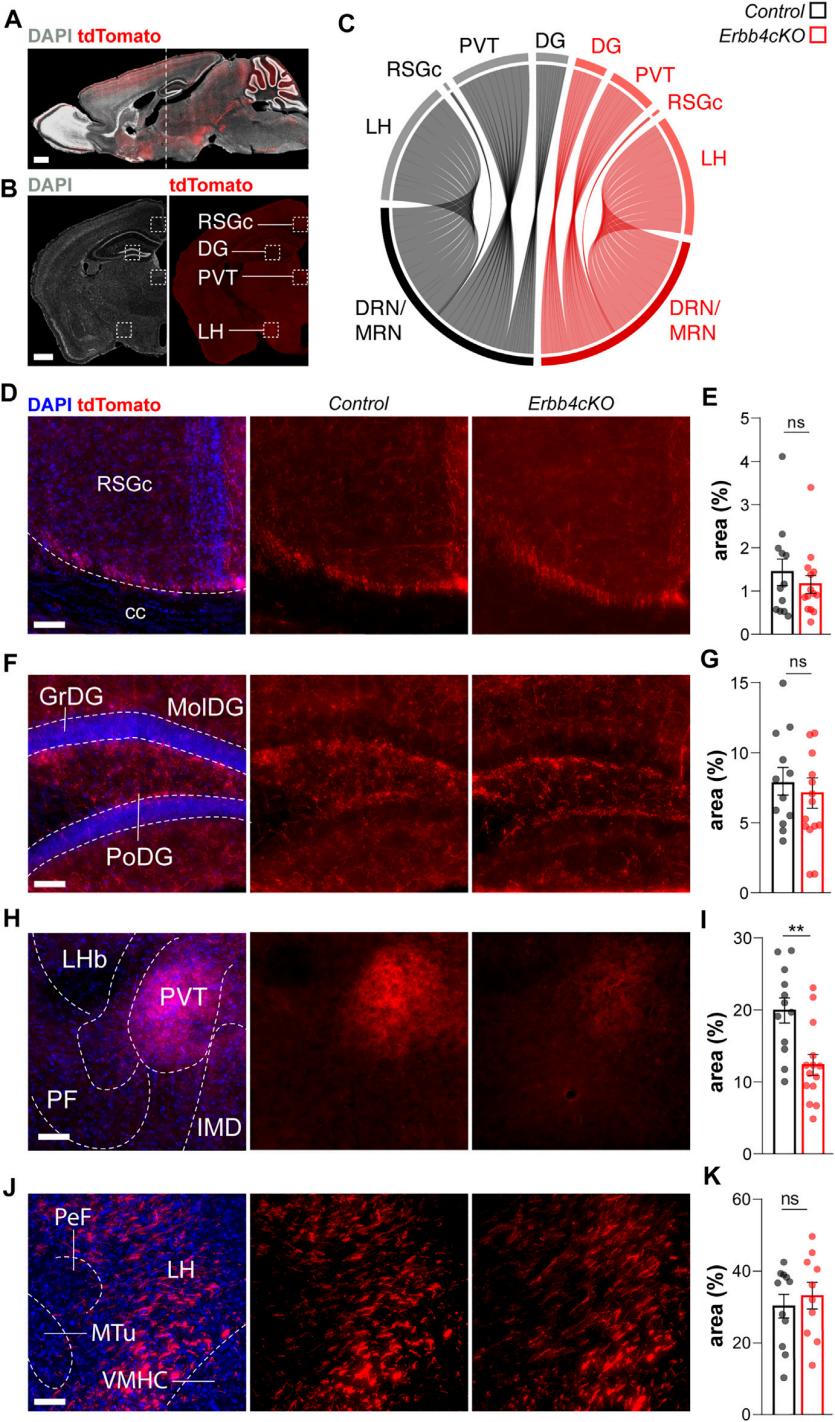
Long-range connectivity deficits in ErbB4-deficient *Pet1*+ neurons of male and female mice. **(A)** *Pet1*+tdTomato+ cells in a sagittal section of a mouse brain. Dashed line indicates the location of the brain regions analysed in coronal sections. **(B)** Coronal section of an adult *Pet1*-reporter mouse showing tdTomato-labelled projections. Dashed squares indicate the location of the brain areas analyzed. **(C)** Circular plot representing relative connectivity to different brain areas from raphe nucleus (RN) in control (black) and *Erbb4cKO* (red) mice. **(D, F, H, J)** Confocal images of *Pet1*+ tdTomato+ connectivity from raphe nucleus to different brain areas for control and *Erbb4cKO* mice. **(E, G, I, K)** Quantification of connectivity area normalized to the field of view are presented as percentage of area occupied by tdTomato signal (area%) (*n* = 11–12 control and *n* = 10–15 mutant from 3 different litters). RSGc: retrosplenial granular cortex c region; cc: corpus callosum; GrDG: granular dentate gyrus; MolDG: molecular dentate gyrus; PoDG: polymorph layer of the dentate gyrus; LHb: lateral habenular nucleus; PVT: periventricular thalamus; PF: parafascicular thalamic nucleus; IMD: intermediodorsal thalamic nucleus; PeF: perifornical nucleus; LH: lateral hypothalamic area; Mtu: medial tuberal nucleus; VMHC: ventromedial hypothalamic nucleus central part. Scales bars in **(A,B)**: 1000 µm and in **(D, F, H, J)**: 100 µm. Data are represented as mean ± SEM. ns: not significant differences; **: *p <* 0.01; t-test or Mann-Whitney non-parametric test.

In addition to migration, NRG/ErbB4 signaling participates in axon navigation and synaptogenesis during embryonic and postnatal development in local and long-range projecting neural circuits within the forebrain (Lopez-Bendito et al., 2006; Fazzari et al., 2010; Del Pino et al., 2013; Del Pino et al., 2017). Therefore, we hypothesized that ErbB4 might be necessary for the development of long-range efferent serotonergic connectivity. To test this hypothesis, we monitored in adult control and *Erbb4cKO* the serotonergic axon arborization across different postsynaptic targets of the serotonergic system: the retrosplenial granular cortex (RSGc), the hippocampus (DG, CA1, and CA3), the posterior periventricular nucleus of the thalamus (PVT) and the lateral hypothalamus (LH) (Figures 3A,B). We observed that serotonergic axons labelled with tdTomato were present in all regions (RSGc, DG, CA3, CA1, PVT, and LH) in control as well as *Erbb4cKO* mice (Figures 3C–J; Supplementary Figure S5). To assess the density of terminal arborization from serotonergic connectivity, we quantified the area occupied by tdTomato+ signal normalized to a field of view/total area in each brain region (Figure 3C). Non-significant differences in axonal density were observed between control and *Erbb4cKO* in RSGs (control: 1.43 ± 0.31%; *Erbb4cKO:* 1.15 ± 0.20%; *p =* 0.446) (Figures 3D,E), hippocampus (control DG: 19.92 ± 1.74%; *Erbb4cKO* DG*:* 12.32 ± 1.44%; *p =* 0.630; control CA3: 1.8 ± 0.2%; *Erbb4cKO* CA3*:* 2.3 ± 0.4%; *p =* 0.509; control CA1: 1.2 ± 0.2%; *Erbb4cKO* CA1*:* 1.3 ± 0.2%; *p =* 0.28; (Figures 3F,G; Supplementary Figure S5); and LH (control: 30.26 ± 3.29%; *Erbb4cKO:* 33.14 ± 3.71%; *p =* 0.568) (Figures 3J,K). In contrast, *Erbb4cKO* mice displayed significantly reduced axonal density in the PVT region when compared to controls (control: 7.8 ± 1.0%; *Erbb4cKO:* 7.1 ± 1.0%; *p =* 0.0025) (Figures 3H,I). These results obtained with epifluorescence imaging were reproduced in the PVT using confocal microscopy (Supplementary Figure S6) (control: 13.5 ± 1.0%; *Erbb4cKO:* 7.5 ± 0.7%; *p =* 0.0002). Thus, our data suggest that, while global serotonergic connectivity arriving to the LH or to other cortical targets (i.e. hippocampus and RSGc) is not dependent on ErbB4 expression, ErbB4 is required for the axonal arborization of a subpopulation of *Pet1*+ serotonergic projections reaching the PVT area.

### 3.4 *Pet1*+ Neurons Lacking Erbb4 Do Not Cause Local Defects in the DRN

**FIGURE 4:**
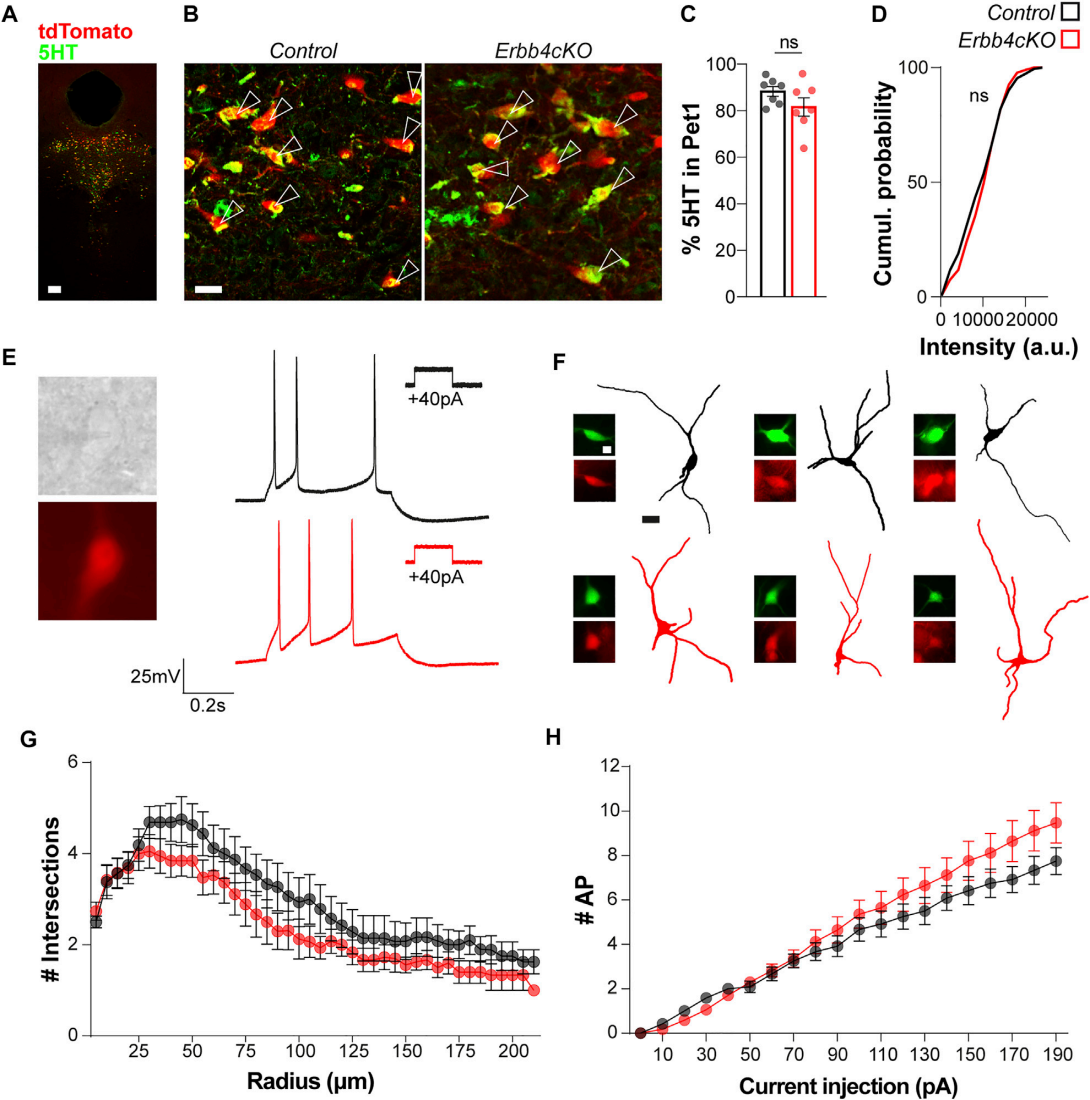
ErbB4 deficiency in Pet + neurons does not lead to changes in global excitability or serotonin content in the DRN in male mice. **(A)** Colocalization of *Pet1*-tdTomato and 5HT in the raphe nucleus. **(B)** Magnifications for the colocalization of *Pet1*-tdTomato and 5HT in control and *Erbb4cKO* mice. Arrow heads point to double positive cells for *Pet1* and 5HT. **(C)** Quantification of the percentage of *Pet1*+ cells expressing 5HT (*n* = 7 control and *n* = 7 mutant mice from 4 different litters). **(D)** Cumulative probability of 5HT intensity for control and *Erbb4cKO* cells expressed as arbitrary units (n = 5 control and *n* = 5 mutant mice from 3 different litters). **(E)** Representative images and traces of *Pet1*+ tdTomato-expressing neurons recorded in current clamp mode in control (black) and *Erbb4cKO* (red) mice. **(F)** Reconstructions of *Pet1*+ cells recorded. Images of streptavidin-Alexa488 (green) and tdTomato (red) for each cell. **(G)** Sholl analysis of *Pet1*+ tdTomato+ cells recorded for control and *Erbb4cKO* mice. Intersections are quantified per radius of 5 µm of difference from soma (*n* = 11 neurons from 3 different control mice and *n* = 16 neurons from 3 different mutant mice). **(H)** Input-output plot showing number of spikes as a function of injected current displayed by *Pet1*+ tdTomato+ neurons in control (*n* = 11 neurons from 3 different control mice) and *Erbb4cKO* mice (*n* = 16 neurons from 3 different mutant mice). Scale bars in **(A)**: 100 and 20 µm and **(F)** 10 and 25 µm. Data are represented as mean ± SEM. ns: not significant differences. t-test, Kolmogorov-Smirnov test or two-way ANOVA with Bonferroni correction for multiple comparisons.

Since serotonergic branching is regulated by serotonin levels during brain development (Migliarini et al., 2013) we investigated whether the serotonergic connectivity deficits observed in the PVT region in *Erbb4cKO* mice could arise from changes in 5HT expression within the DRN. Immunohistochemistry against 5HT and colocalization analysis between 5HT+ and tdTomato+ cells did not reveal significant differences between the number of *Pet1*+ neurons expressing 5HT in controls and *Erbb4cKO* mice (control: 88.29 ± 3.90%; *Erbb4cKO:* 81.55 ± 2.11; *p = 0.155*) (Figures 4A–C). Quantification of 5HT signal intensity measured within *Pet1*+ neurons followed a similar distribution in controls and *Erbb4cKO* mice (*p > 0.05; Kolmogorov-Smirnov test*) (Figure 4D), indicating that ErbB4 deletion does not affect global 5HT content in the DRN.

To further understand whether developmental deletion of ErbB4 in serotonergic cells alters the excitability of the DRN, we probed the intrinsic electrophysiological properties of tdTomato+ neurons in acute brain slices of controls and *Erbb4cKO* mice. Through whole-cell patch-clamp recordings performed in adult tdTomato+ neurons within the DRN (Figure 4E), we observed that the majority of neurons exhibited spike frequency adaptation (73% in control [11 of 15 recorded cells]; 84% in mutant [16 of 19 recorded cells]). For comparison of intrinsic properties, we explored whether neurons recorded belonged to a similar neural subtype. We examined the morphology of neurons recorded by performing *post hoc* histochemistry against biocytin-filled tdTomato+ neurons. Sholl analysis of neuronal morphology did not reveal significant differences between control and *Erbb4cKO* mice (*p* > 0.05; *two-way ANOVA test*) (Figures 4F,G), indicating that recorded neurons with adapting firing profile displayed similar dendrite complexity and suggesting that they belonged to an electrophysiologically and morphologically similar *Pet1*-expressing neuronal subtype. Next, our analysis of multiple electrophysiological properties as well as the input/output relationship did not reveal significant differences in most of the passive and active intrinsic properties between neurons from control and *Erbb4cKO* mice (*p* > 0.05; *two-way ANOVA test*) (Figure 4H; Table 1). A significantly reduced medium after hyperpolarization was observed in *Erbb4cKO* mice when compared to control (Table 1), indicating that ErbB4 deficiency in serotonergic neuron has an effect on specific membrane properties without causing a prominent change in global excitability. Altogether, our findings suggest that ErbB4 dysfunction in the DRN does not lead to a local phenotype in DRN neither at the intrinsic excitability level nor in serotonergic tone of *Pet1*+ neurons.

**TABLE 1:** Global intrinsic excitability of *Pet1*+tdTomato+ neurons in the dorsal raphe is not affected by ErbB4-deficiency in serotonergic neurons. Intrinsic electrophysiological properties of *Pet1*+ tdTomato-expressing neurons in the dorsal raphe nucleus of control and *Erbb4cKO* mice. V_*rest*_: resting membrane potential; R_*in*_: input resistance; AHP: afterhyperpolatization; FS latency: latency to first spike at rheobase; MFF: maximum firing frequency. Data are represented as mean ± SEM.

	*Ctrl*	*Erbb4cKO*	*p-value*	Test
RMP	−67.21 ± 2.104	−63.02 ± 1.546	0.1133	t-test
R*in* (MΩ)	1,053 ± 125.2	1,189 ± 108.7	0.4237	t-test
Amplitude (mV)	71.79 ± 1.480	67.66 ± 2.718	0.5437	Mann-Whitney
Threshold (mV)	−29.45 ± 1.2460	−30.83 ± 1.846	0.5811	t-test
Rise Time (ms)	0.8264 ± 0.03684	1.130 ± 0.3047	0.6709	Mann-Whitney
Decay Time (ms)	3.601 ± 0.2138	3.224 ± 0.1676	0.1691	t-test
Half-width (ms)	2.990 ± 0.1995	2.636 ± 01532	0.1646	t-test
fAHP (ms)	−19.13 ± 1.221	−21.10 ± 1.153	0.2626	t-test
mAHP (ms)	−21.32 ± 1.085	−17.49 ± 1.308	0.0486(*)	t-test
Rheobase (pA)	17.27 ± 2.727	25.00 ± 2.739	0.0664	Mann-Whitney
FS latency (ms)	193.4 ± 19.70	182.9 ± 22.09	0.7414	t-test
Inter-spike interval (ms)	289.2 ± 28.29	236.0 ± 23.00	0.1555	t-test
Spike Frequency Accommodation	0.29 ± 0.02	0.42 ± 0.75	0.1635	t-test
MFF (Hz)	4.00 ± 0.30	4.78 ± 0.48	0.2275	t-test

### 3.5 ErbB4 Deficiency in *Pet1*+ Serotonergic Neurons Cause Specific Behavioral Impairments in Social and Fear Memory

**FIGURE 5:**
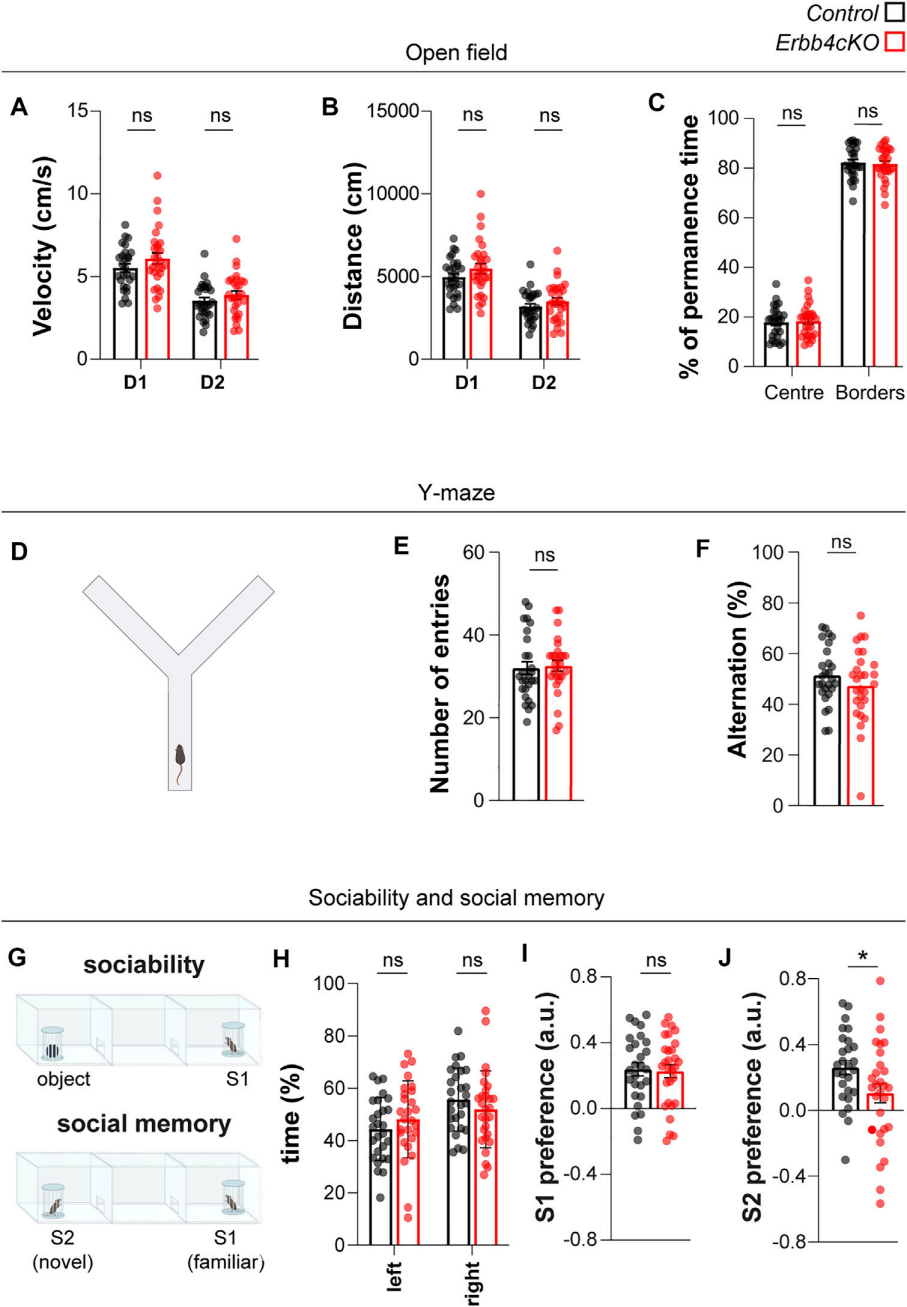
Mice with Erbb4 deletion in *Pet1*+ neurons display social memory deficits but no phenotypes on sociability, locomotor activity or attentional levels. **(A–C)** Locomotor activity in the open field during day 1 (D1) and day 2 (D2) of control and *Erbb4cKO* mice is represented as velocity **(A)** and distance travelled **(B)**. **(C)** Percentage of permanence time in the centre and borders of the open field. **(D,E)** Locomotor activity was measured in the Y-maze as total number of entries. **(F)** Working memory measured as percentage of spontaneous alternations during Y-maze test. **(G)** Schematic of the sociability test and social novelty test. **(H)** Percentage of time spent in the lateral chambers during the habituation phase to the 3-chamber test. **(I)** Index of preference of S1 versus object during sociability phase. **(J)** Index of preference of S2 versus S1 during social memory phase. In the statistical analysis of all experiments described in this figure, the sex perspective has been included. Animals of both sexes were included in the samples of each experiment. After checking that patterns of data distribution were common in males and females via a stratified analysis by sex, pooled analysis was carried out. For all these behavioral tests, *n* = 27 (11 females, 16 males) control and *n* = 29 (12 females, 17 males) mutant from 7 different litters. Data are represented as mean ± SEM. ns: not significant differences; *: *p < 0.*05. t-test, Mann-Whitney or two-way ANOVA with Bonferroni correction for multiple comparisons.

To evaluate whether the connectivity deficits observed upon ErbB4-deficiency in *Pet1*+ serotonergic neurons impact on brain function, we tested control (*Erbb4* ^*f/f*^) and *Erbb4cKO* littermates in behavioral assays. Spontaneous locomotor activity was evaluated using the open field and the Y-maze, respectively. Controls and *Erbb4cKO* mice travelled similar distances (control: 49.63 ± 2.22 m; *Erbb4cKO:* 54.77 ± 301 m; *p =* 0.254) with a comparable velocity in the open field (control: 5.52 ± 0.24 cm/s; *Erbb4cKO:* 6.09 ± 0.33 cm/s; *p =* 0.255) (Figures 5A,B). In addition, we did not find differences in other parameters indicative of anxiety such as the time spent in the center *vs* borders in the open field between control and mutant mice (Centre: control: 17.81 ± 1.24%; *Erbb4cKO:* 18.36 ± 1.207%; *p* > 0,9999; Borders: control: 82.19 ± 1.24%; *Erbb4cKO:* 81.64 ± 1.21%; *p* > 0.9999) (Figure 5C). Spontaneous alternation was also evaluated in the Y-maze (Figures 5D–F). Similar number of entries (control: 32.00 ± 1.55; *Erbb4cKO:* 32.54 ± 1.30; *p =* 0.792) (Figure 5E) as well as percent of alternations were observed between control and *Erbb4cKO* mice (control: 51.40 ± 2.19%; *Erbb4cKO:* 47.25 ± 2.71; *p =* 0.242) (Figure 5F), indicating that ErbB4-dysfunction in the serotonergic systems does not affect locomotor activity or working memory.

To test sociability and social memory we employed the three-chamber test that consisted of three sessions: habituation (empty cylinders), sociability (conspecific mouse “stranger 1” or “S1” and object) and preference for social novelty (familiar mouse “S1” and new-conspecific “stranger 2” or “S2”) (Figure 5G). In the habituation phase, mice explored similarly the lateral chambers of the 3-chamber test (Figure 5H), indicating no contextual preference. During the sociability phase, we observed no differences between control and *Erbb4cKO* mice in distance travelled (data not shown), in agreement with a lack of locomotion phenotype in the open field. Both genotypes showed preference for mouse S1 over the object (control: 0.23 ± 0.040; *Erbb4cKO:* 0.22 ± 0.04; *p =* 0.8353) (Figure 5I). However, when animals were exposed to the preference for social novelty test, we observed that *Erbb4cKO* mice spent less time exploring the novel mouse S2 and displayed a significantly reduced preference index for the novel S2 conspecific over the previously known S1 mouse when compared to controls (control: 0.26 ± 0.04; *Erbb4cKO:* 0.10 ± 0.06; *p =* 0.036) (Figure 5J). Together, the lack of sociability phenotype and the reduced preference for social novelty that we observed suggest that social memory could be impaired in *Erbb4cKO* mice.

**FIGURE 6:**
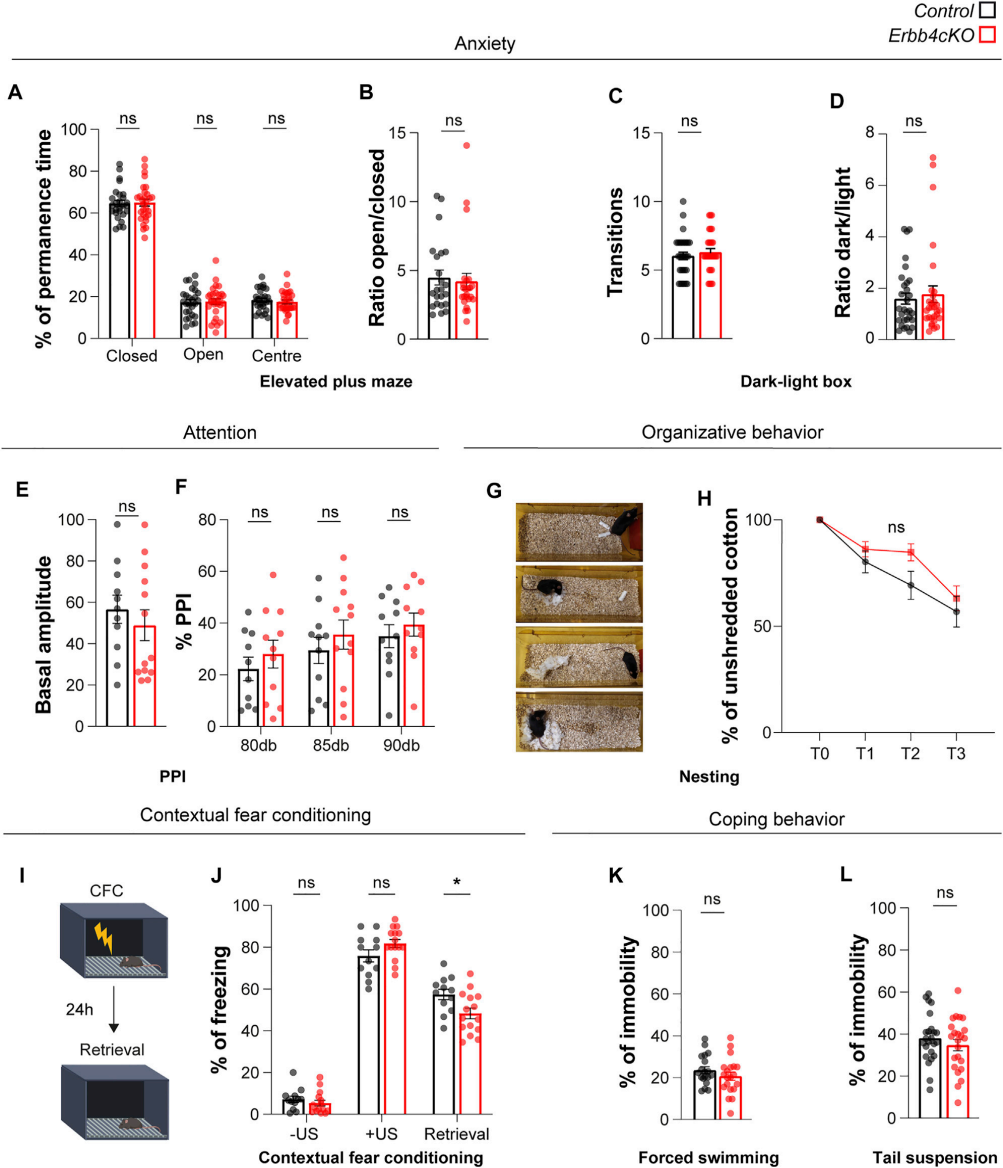
Mice with Erbb4 deletion in *Pet1*+ neurons display fear memory deficits but do not show abnormalities in anxiety or organizational behavior. **(A)** Percentage of permanence time the closed arms, open arms and centre of the elevated plus maze test. **(B)** Ratio of permanence time in the open arms over the closed arms during the elevated plus maze test. **(C)** Number of transitions between lighted and dark areas in the light-dark box test **(D)** Ratio between dark and light permanence time in light-dark box test. **(E)** Basal amplitude of startle reflex for control and *Erbb4cKO* mice. **(F)** Percentage of prepulse inhibition (PPI) of startle response for control and *Erbb4cKO* mice. **(G)** Sample images of four different time points during the nest building test. **(H)** Percentage of unshredded cotton used by mice for building a nest at 0, 1, 2, and 3 h after providing them with new material. **(I)** Diagram of contextual fear conditioning (CFC) test (mofidied from Biorender.com). Retrieval test was performed 24 h after CFC by placing the mouse in the same context. **(J)** Percentage of freezing for each phase of the CFC learning and retrieval. -US: before unconditioned stimulus; +US: after unconditioned stimulus; Retrieval: 1 day after CFC. **(K)** Percentage of immobility for the forced swimming test. **(L)** Percentage of immobility in the tail suspension test. In the statistical analysis of all experiments described in this figure, the sex perspective has been included. Animals of both sexes were included in the samples of each experiment. After checking that patterns of data distribution were common in males and females via a stratified analysis by sex, pooled analysis was carried out. For all behavioral tests except PPI, CFC, forced swimming and tail suspension, *n* = 27 (11 females, 16 males) control and *n* = 29 (12 females, 17 males) mutant mice from 8 different litters. For PPI test, *n* = 11 (6 females, 5 males) control and *n* = 13 (8 females, 5 males) mutant mice from 4 different litters. For CFC test, *n* = 12 (7 females, 5 males) control and *n* = 15 (9 females, 6 males) mutant from 4 different litters. For forced swimming, *n* = 19 (7 females, 12 males) control and *n* = 20 (7 females, 13 males) mutant mice. For tail suspension, *n* = 24 (13 females, 11 males) and *n* = 23 (13 females, 10 males). Data are represented as mean ± SEM. ns: not significant differences; *: *p* < 0.05. t-test, Mann-Whitney or two-way ANOVA with Bonferroni correction for multiple comparisons.

To elucidate whether alterations found in social memory might be influenced by anxiety, control and *Erbb4cKO* animals were tested for anxiety-like behavior using the elevated plus maze and the dark-light box test. In agreement with a lack of anxiety-like behavior in the open field (Figure 5C), we did not find significant differences between control and mutant mice in the percentage of time spent in the different zones of the elevated plus maze (Figure 6A), or in the ratio of time spent in open vs close arms of the elevated plus maze (control: 4.48 ± 0.534; *Erbb4cKO:* 4.20 ± 0.59; *p =* 0.729) (Figure 6B). In addition, we did not observe significant differences in the number of transitions (control: 6.0 ± 0.27; *Erbb4cKO*: 6.30 ± 0.26; *p =* 0.448) (Figure 6C) and ratio of time spent in the dark over the brightly illuminated area (control: 1.60 ± 0.21; *Erbb4cKO:* 1.77 ± 0.32; *p =* 0.660) in the dark*-*light box test (Figure 6D), indicating that Erbb4 deficiency in *Pet1*+ serotonergic circuits have no effect on anxiety levels.

To further elucidate whether behavioral deficits could involve abnormal attentional levels, control and *Erbb4cKO* animals were tested in the prepulse inhibition of the startle reflex (PPI) test. Both genotypes display startle reflex to a 120dB sound (control: 56.64 ± 6.81%; *Erbb4cKO*: 48.89 ± 7.45%; *p* = 0.457) (Figure 6E). Inhibition of startle reflex by the presentation of a prepulse of 80, 85 or 90 dB was not significantly different between controls and *Erbb4cKO* animals (80dB: control: 18.67 ± 5.46%; *Erbb4cKO*: 22.95 ± 5.72%; *p* > 0.999; 85dB: control: 29.43 ± 5.07%; *Erbb4cKO*: 32.37 ± 6.09%; *p* > 0.999; 90 dB: control: 34.97 ± 4.49%; *Erbb4cKO*: 33.04 ± 5.71%; *p* > 0.999) (Figure 6F), suggesting that *Erbb4cKO* do not have attentional deficits.

We also monitored nesting behavior in the home cage to determine the species-typical behavior of *Erbb4cKO* animals (Figure 6G). We did not observe significant differences in the ability of animals to build a nest between controls and *Erbb4cKO* mice, suggesting that serotonergic deficits observed in *Erbb4cKO* mice are not relevant for the innate organized behavior (*p > 0.05; two-way ANOVA*) (Figure 6H).

In light of the deficits observed in social memory in *Erbb4cKO* animals, we evaluated whether other types of memory could be affected by Erbb4 deficiency in *Pet1*+ serotonergic circuits with the contextual fear conditioning paradigm (Figure 6I). While *Erbb4cKO* and control mice displayed similar levels of freezing upon presentation of the unconditional stimulus (electric shock), freezing displayed 24 h after fear conditioning during fear memory retrieval was significantly reduced in *Erbb4cKO* mice when compared to controls (control: 57.37 ± 2.49; *Erbb4cKO:* 48.28 ± 2.54; *p =* 0.013) (Figure 6J). These data indicated that fear memory retrieval is impaired in *Erbb4cKO* mice.

Finally, to assess whether Erbb4-deficiency in *Pet1*+ neurons is affecting parallel serotonergic sub-systems described to regulate coping behavior in face of challenge (Ren et al., 2018), mice performed the forced swimming and tail suspension tasks. In both tasks, *Erbb4cKO* mutants and control mice presented similar levels of immobility (FS: control: 23.57 ± 1.64s; *Erbb4cKO:* 20.66 ± 1.95s; *p =* 0.263; TS: control: 38.03 ± 2.37s; *Erbb4cKO:* 34.75 ± 2.66s; *p =* 0.361) (Figures 6K–L) indicative of normal coping behavior in *Erbb4cKO* mutants.

Altogether our data suggest that developmental deficiency of *Erbb4* in *Pet1*+ serotonergic circuits leads to the abnormal processing of specific types of memory that contain a social or aversive components, but does not have an effect on locomotion, emotional, attentional or organizational behaviors.

## 4 Discussion

Here, we provide evidence for a new role of ErbB4 in the development of serotonergic sub-systems. In particular, we found that long-range connectivity taking place between *Pet1*-expressing subpopulations and the PVT area requires ErbB4. In addition, our behavioral characterization revealed that the developmental disruption of ErbB4 in discrete serotonergic neurons has selective functional consequences on the processing of specific types of memory associated with an emotional or social component, such as fear and social memory.

The formation of serotonergic axons takes place over a protracted period during brain development spanning embryonic to postnatal stages. In rodents, axon growth begins early after serotonergic neuron migration reaching target areas by birth (Bang et al., 2012). Subsequently, terminal arborization sprouting and refinement takes place during the postnatal period (Lidov and Molliver, 1982; Maddaloni et al., 2017). Here we show that NRG/ErbB4 signaling is necessary for the proper arborization of serotonergic axons reaching the PVT, but is dispensable for the innervation of other targets of the serotonergic system (lateral hypothalamus, hippocampus and retrosplenial cortex). Previous work demonstrates that more than one subgroup of serotonergic subpopulations from different rhombomeric segments innervates an individual target area (Bang et al., 2012). In particular, it was reported that the PVT is innervated by Pet1-expressing serotonergic neurons originating from rhombomere (r)1 — populating the DRN, MRN and B9 groups — and from r2 — populating the MRN exclusively — (Jensen et al., 2008; Bang et al., 2012; Senft et al., 2021), but we can not exclude that other raphe nuclei also project to the PVT region. It remains to be determined whether our *Erbb4cKO* approach disrupts axon arborization from ErbB4 expressing neurons that are both in the DRN and MRN. Our results demonstrate that serotonergic circuit formation of long-range projections targeting the PVT is regulated by NRG/ErbB4 signaling. However, we can not exclude that: 1) the PVT could be also innervated by ErbB4-negative serotonergic neurons and 2) that disruption of ErbB4 in serotonergic circuits affects axon development in other postsynaptic targets not visited in this study. While it is reasonable to consider that serotonergic circuit wiring relies on the combination of guidance and signaling molecules as well as environmental factors during development, our findings reinforce the notion that intrinsic signaling pathways regulated by ErbB4, act as organizers of specific serotonergic sub-systems during brain development.

The developmental disruption of ErbB4 in serotonergic circuits has a very specific impact on the behavioral performance of mice. The specific behavioral phenotypes restricted to fear and social memory deficits observed in *Erbb4* ^*f/f;Pet1-Cre;Ai9f/*+^ mice are in line with the participation of NRG/ErbB4 signaling in the development of discrete serotonergic circuits. The notion that serotonergic circuits are organized into segregated sub-systems regulating specific behaviors (Abrams et al., 2004) is supported by functional manipulations of projection-defined serotonergic circuits. For example, studies show that specific long-range efferent projections from the DRN play a role in anxiety [DRN → bed nucleus of the stria terminalis (Marcinkiewcz et al., 2016), DRN → central amygdala (Ren et al., 2018)], coping behavior [DRN → orbitofrontal cortex (Ren et al., 2018)], sociability [DRN → anterior cingulate cortex (Li et al., 2021)] and fear memory [DRN → bed nucleus of the stria terminalis (Marcinkiewcz et al., 2016)]. Other excellent studies inform about serotonergic sub-systems characterized by dopamine receptor expression modulating aggressive behavior through multiple postsynaptic targets (Niederkofler et al., 2016). Our findings disclose a serotonergic sub-system that relies on ErbB4 for proper regulation of at least two types of memory: associative fear memory and social memory. Interestingly, several studies link the function of PVT to fear memory processing in rodents (Padilla-Coreano et al., 2012; Li et al., 2014; Do-Monte et al., 2015; Penzo et al., 2015; Choi and McNally, 2017; Chen and Bi, 2019) and sociability (Yamamuro et al., 2020). Although different types of neurons within the PVT have been shown to regulate emotional and motivated behaviors, our study is the first to report the association between circuits integrating the PVT and the formation of social memories.

Recently, a study showed that downregulation of ErbB4 in adult mice results in hyperexcitability of serotonergic neurons and anxiogenic behavior (Zhang et al., 2020). In contrast, we show that the developmental disruption of ErbB4 does not cause an anxiogenic response in mice nor it leads to prominent changes in global excitability of serotonergic neurons. Hence, the functional implications of acute downregulation of *Erbb4* in the adult brain are very different from the ErbB4-deficiency in serotonergic circuits during its development. We deduce this could be due to unknown developmental plasticity rules in the serotonergic system, that are absent in the adult brain. Furthermore, the different outcomes obtained from adult and developmental *Erbb4* disruption might result from the genetic targeting of different serotonergic subsets, since the study of Zhang et al. (2020) employed the *Sert-Cre* mice to target *Erbb4* expression in the adult brain and our data suggests that subsets of 5HT+ErbB4+ neurons are *Pet1*-negative in the DRN and MRN.

Finally, substantial evidence associate Nrg/Erbb4 signaling to neurodevelopmental disorders (reviewed in(Mei and Nave, 2014)) such as schizophrenia (Norton et al., 2006; Silberberg et al., 2006; Walsh et al., 2008; Mostaid et al., 2017). Most of our knowledge on cognitive dysfunction in NRG/ErbB4-deficiency disorders stems from studies focusing on cortical GABAergic interneurons, which are the most numerous neuronal subtype expressing ErbB4 in the brain (Fazzari et al., 2010) (reviewed in (Rico and Marin, 2011)). Our findings reveal novel neural circuit basis (i.e. DRN/MRN → PVT) of cognitive dysfunction in NRG/ErbB4-deficiency disorders involving GABAergic and non-GABAergic neurons in the raphe nuclei. In addition to the already known defects in cortical inhibitory/excitatory balance (Del Pino et al., 2013; Del Pino et al., 2017; Wang et al., 2018) and in the dopaminergic system (Skirzewski et al., 2018), our results on DRN → PVT circuit dysfunction contribute to better understand the pathophysiology of ErbB4-deficiency disorders.

Altogether, we disclose ErbB4 as new intrinsic factor of serotonergic subpopulations that acts as organizer of 5HT long-range sub-circuits regulating the formation of emotional and social memories.

## Data Availability

The original contributions presented in the study are included in the article/Supplementary Material, further inquiries can be directed to the corresponding author.

## References

[B1] Abrams J. K. Johnson P. L. Hollis J. H. Lowry C. A. (2004). Anatomic and Functional Topography of the Dorsal Raphe Nucleus. Ann. N Y Acad. Sci. 1018, 46–57. 10.1196/annals.1296.005 15240351

[B2] Arshadi C. Günther U. Eddison M. Harrington K. I. S. Ferreira T. A. (2021). SNT: a Unifying Toolbox for Quantification of Neuronal Anatomy. Nat. Methods 18, 374–377. 10.1038/s41592-021-01105-7 33795878

[B3] Bang S. J. Jensen P. Dymecki S. M. Commons K. G. (2012). Projections and Interconnections of Genetically Defined Serotonin Neurons in Mice. Eur. J. Neurosci. 35, 85–96. 10.1111/j.1460-9568.2011.07936.x 22151329 PMC3268345

[B4] Bartolini G. Sánchez-Alcañiz J. A. Osório C. Valiente M. García-Frigola C. Marín O. (2017). Neuregulin 3 Mediates Cortical Plate Invasion and Laminar Allocation of GABAergic Interneurons. Cel Rep. 18, 1157–1170. 10.1016/j.celrep.2016.12.089 PMC5300889 28147272

[B5] Batista-Brito R. Vinck M. Ferguson K. A. Chang J. T. Laubender D. Lur G. (2017). Developmental Dysfunction of VIP Interneurons Impairs Cortical Circuits. Neuron 95, 884–895. 10.1016/j.neuron.2017.07.034 28817803 PMC5595250

[B6] Bean J. C. Lin T. W. Sathyamurthy A. Liu F. Yin D.-M. Xiong W.-C. (2014). Genetic Labeling Reveals Novel Cellular Targets of Schizophrenia Susceptibility Gene: Distribution of GABA and Non-GABA ErbB4-Positive Cells in Adult Mouse Brain. J. Neurosci. 34, 13549–13566. 10.1523/JNEUROSCI.2021-14.2014 25274830 PMC4180480

[B7] Chen M. Bi L.-l. (2019). Optogenetic Long-Term Depression Induction in the PVT-CeL Circuitry Mediates Decreased Fear Memory. Mol. Neurobiol. 56, 4855–4865. 10.1007/s12035-018-1407-z 30406427

[B8] Choi E. A. McNally G. P. (2017). Paraventricular Thalamus Balances Danger and Reward. J. Neurosci. 37, 3018–3029. 10.1523/JNEUROSCI.3320-16.2017 28193686 PMC6596734

[B9] Dahlström A. Fuxe K. (1964). Localization of Monoamines in the Lower Brain Stem. Experientia 20, 398–399. 10.1007/BF02147990 5856530

[B10] Del Pino I. Brotons-Mas J. R. Marques-Smith A. Marighetto A. Frick A. Marín O. (2017). Abnormal Wiring of CCK+ Basket Cells Disrupts Spatial Information Coding. Nat. Neurosci. 20, 784–792. 10.1038/nn.4544 28394324 PMC5446788

[B11] del Pino I. García-Frigola C. Dehorter N. Brotons-Mas J. R. Alvarez-Salvado E. Martínez de Lagrán M. (2013). Erbb4 Deletion from Fast-Spiking Interneurons Causes Schizophrenia-like Phenotypes. Neuron 79, 1152–1168. 10.1016/j.neuron.2013.07.010 24050403

[B12] Do-Monte F. H. Quiñones-Laracuente K. Quirk G. J. (2015). A Temporal Shift in the Circuits Mediating Retrieval of Fear Memory. Nature 519, 460–463. 10.1038/nature14030 25600268 PMC4376623

[B13] Donovan L. J. Spencer W. C. Kitt M. M. Eastman B. A. Lobur K. J. Jiao K. (2019). Lmx1b Is Required at Multiple Stages to Build Expansive Serotonergic Axon Architectures. Elife 8, 1. 10.7554/eLife.48788 PMC6685705 31355748

[B14] Fazzari P. Paternain A. V. Valiente M. Pla R. Luján R. Lloyd K. (2010). Control of Cortical GABA Circuitry Development by Nrg1 and ErbB4 Signalling. Nature 464, 1376–1380. 10.1038/nature08928 20393464

[B15] Fernandez S. P. Cauli B. Cabezas C. Muzerelle A. Poncer J.-C. Gaspar P. (2016). Multiscale Single-Cell Analysis Reveals Unique Phenotypes of Raphe 5-HT Neurons Projecting to the Forebrain. Brain Struct. Funct. 221, 4007–4025. 10.1007/s00429-015-1142-4 26608830

[B16] Flames N. Long J. E. Garratt A. N. Fischer T. M. Gassmann M. Birchmeier C. (2004). Short- and Long-Range Attraction of Cortical GABAergic Interneurons by Neuregulin-1. Neuron 44, 251–261. 10.1016/j.neuron.2004.09.028 15473965

[B17] Forero A. Rivero O. Wäldchen S. Ku H.-P. Kiser D. P. Gärtner Y. (2017). Cadherin-13 Deficiency Increases Dorsal Raphe 5-HT Neuron Density and Prefrontal Cortex Innervation in the Mouse Brain. Front. Cel. Neurosci. 11, 307. 10.3389/fncel.2017.00307 PMC5623013 29018333

[B18] Franklin K. B. J. Paxinos G. (2008). The Mouse Brain in Stereotaxic Coordinates, Compact. 3rd ed.. USA: Elsevier Academic Press.

[B19] Garcia S. Guarino D. Jaillet F. Jennings T. Pröpper R. Rautenberg P. L. (2014). Neo: an Object Model for Handling Electrophysiology Data in Multiple Formats. Front. Neuroinform. 8, 10. 10.3389/fninf.2014.00010 24600386 PMC3930095

[B20] Golub M. S. Germann S. L. Lloyd K. C. K. (2004). Behavioral Characteristics of a Nervous System-specific erbB4 Knock-Out Mouse. Behav. Brain Res. 153, 159–170. 10.1016/j.bbr.2003.11.010 15219717

[B21] Hawthorne A. L. Wylie C. J. Landmesser L. T. Deneris E. S. Silver J. (2010). Serotonergic Neurons Migrate Radially through the Neuroepithelium by Dynamin-Mediated Somal Translocation. J. Neurosci. 30, 420–430. 10.1523/JNEUROSCI.2333-09.2010 20071506 PMC2855244

[B22] Hay-Schmidt A. (2000). The Evolution of the Serotonergic Nervous System. Proc. R. Soc. Lond. B 267, 1071–1079. 10.1098/rspb.2000.1111 PMC1690648 10885511

[B23] Hendricks T. Francis N. Fyodorov D. Deneris E. S. (1999). The ETS Domain Factor Pet-1 Is an Early and Precise Marker of central Serotonin Neurons and Interacts with a Conserved Element in Serotonergic Genes. J. Neurosci. 19, 10348–10356. 10.1523/jneurosci.19-23-10348.1999 10575032 PMC6782418

[B24] Jensen P. Farago A. F. Awatramani R. B. Scott M. M. Deneris E. S. Dymecki S. M. (2008). Redefining the Serotonergic System by Genetic Lineage. Nat. Neurosci. 11, 417–419. 10.1038/nn2050 18344997 PMC2897136

[B25] La Manno G. Siletti K. Furlan A. Gyllborg D. Vinsland E. Mossi Albiach A. (2021). Molecular Architecture of the Developing Mouse Brain. Nature 596, 92–96. 10.1038/s41586-021-03775-x 34321664

[B26] Li L. Zhang L.-Z. He Z.-X. Ma H. Zhang Y.-T. Xun Y.-F. (2021). Dorsal Raphe Nucleus to Anterior Cingulate Cortex 5-HTergic Neural Circuit Modulates Consolation and Sociability. Elife 10, 1. 10.7554/eLife.67638 PMC8213405 34080539

[B27] Li Y. Dong X. Li S. Kirouac G. J. (2014). Lesions of the Posterior Paraventricular Nucleus of the Thalamus Attenuate Fear Expression. Front. Behav. Neurosci. 8, 94. 10.3389/fnbeh.2014.00094 24688461 PMC3960725

[B28] Lidov H. G. W. Molliver M. E. (1982). An Immunohistochemical Study of Serotonin Neuron Development in the Rat: Ascending Pathways and Terminal fields. Brain Res. Bull. 8, 389–430. 10.1016/0361-9230(82)90077-6 6178481

[B29] López-Bendito G. Cautinat A. Sánchez J. A. Bielle F. Flames N. Garratt A. N. (2006). Tangential Neuronal Migration Controls Axon Guidance: a Role for Neuregulin-1 in Thalamocortical Axon Navigation. Cell 125, 127–142. 10.1016/j.cell.2006.01.042 16615895 PMC2365888

[B30] Maddaloni G. Bertero A. Pratelli M. Barsotti N. Boonstra A. Giorgi A. (2017). Development of Serotonergic Fibers in the Post-Natal Mouse Brain. Front. Cel. Neurosci. 11, 202. 10.3389/fncel.2017.00202 PMC5509955 28769763

[B31] Madisen L. Zwingman T. A. Sunkin S. M. Oh S. W. Zariwala H. A. Gu H. (2010). A Robust and High-Throughput Cre Reporting and Characterization System for the Whole Mouse Brain. Nat. Neurosci. 13, 133–140. 10.1038/nn.2467 20023653 PMC2840225

[B32] Marcinkiewcz C. A. Mazzone C. M. D’Agostino G. Halladay L. R. Hardaway J. A. DiBerto J. F. (2016). Serotonin Engages an Anxiety and Fear-Promoting Circuit in the Extended Amygdala. Nature 537, 97–101. 10.1038/nature19318 27556938 PMC5124365

[B33] Mei L. Nave K.-A. (2014). Neuregulin-ERBB Signaling in the Nervous System and Neuropsychiatric Diseases. Neuron 83, 27–49. 10.1016/j.neuron.2014.06.007 24991953 PMC4189115

[B34] Migliarini S. Pacini G. Pelosi B. Lunardi G. Pasqualetti M. (2013). Lack of Brain Serotonin Affects Postnatal Development and Serotonergic Neuronal Circuitry Formation. Mol. Psychiatry 18, 1106–1118. 10.1038/mp.2012.128 23007167

[B35] Mostaid M. S. Mancuso S. G. Liu C. Sundram S. Pantelis C. Everall I. P. (2017). Meta-analysis Reveals Associations between Genetic Variation in the 5′ and 3′ Regions of Neuregulin-1 and Schizophrenia. Transl Psychiatry 7, e1004. 10.1038/tp.2016.279 28094814 PMC5545738

[B36] Niederkofler V. Asher T. E. Okaty B. W. Rood B. D. Narayan A. Hwa L. S. (2016). Identification of Serotonergic Neuronal Modules that Affect Aggressive Behavior. Cel Rep. 17, 1934–1949. 10.1016/j.celrep.2016.10.063 PMC5156533 27851959

[B37] Norton N. Moskvina V. Morris D. W. Bray N. J. Zammit S. Williams N. M. (2006). Evidence that Interaction between Neuregulin 1 and its Receptor erbB4 Increases Susceptibility to Schizophrenia. Am. J. Med. Genet. 141B, 96–101. 10.1002/ajmg.b.30236 16249994

[B38] Okaty B. W. Freret M. E. Rood B. D. Brust R. D. Hennessy M. L. deBairos D. (2015). Multi-Scale Molecular Deconstruction of the Serotonin Neuron System. Neuron 88, 774–791. 10.1016/j.neuron.2015.10.007 26549332 PMC4809055

[B39] Okaty B. W. Sturrock N. Escobedo Lozoya Y. Chang Y. Senft R. A. Lyon K. A. (2020). A Single-Cell Transcriptomic and Anatomic Atlas of Mouse Dorsal Raphe *Pet1* Neurons. Elife 9, 1. 10.7554/eLife.55523 PMC7308082 32568072

[B40] Padilla-Coreano N. Do-Monte F. H. Quirk G. J. (2012). A Time-dependent Role of Midline Thalamic Nuclei in the Retrieval of Fear Memory. Neuropharmacology 62, 457–463. 10.1016/j.neuropharm.2011.08.037 21903111 PMC3195904

[B41] Penzo M. A. Robert V. Tucciarone J. De Bundel D. Wang M. Van Aelst L. (2015). The Paraventricular Thalamus Controls a central Amygdala Fear Circuit. Nature 519, 455–459. 10.1038/nature13978 25600269 PMC4376633

[B42] Ren J. Friedmann D. Xiong J. Liu C. D. Ferguson B. R. Weerakkody T. (2018). Anatomically Defined and Functionally Distinct Dorsal Raphe Serotonin Sub-systems. Cell 175, 472–487. e420. 10.1016/j.cell.2018.07.043 30146164 PMC6173627

[B43] Ren J. Isakova A. Friedmann D. Zeng J. Grutzner S. M. Pun A. (2019). Single-cell Transcriptomes and Whole-Brain Projections of Serotonin Neurons in the Mouse Dorsal and Median Raphe Nuclei. Elife 8, 1. 10.7554/eLife.49424 PMC6812963 31647409

[B44] Rico B. Marín O. (2011). Neuregulin Signaling, Cortical Circuitry Development and Schizophrenia. Curr. Opin. Genet. Dev. 21, 262–270. 10.1016/j.gde.2010.12.010 21295966

[B45] Scott M. M. Wylie C. J. Lerch J. K. Murphy R. Lobur K. Herlitze S. (2005). A Genetic Approach to Access Serotonin Neurons for *In Vivo* and *In Vitro* Studies. Proc. Natl. Acad. Sci. 102, 16472–16477. 10.1073/pnas.0504510102 16251278 PMC1283423

[B46] Senft R. A. Freret M. E. Sturrock N. Dymecki S. M. (2021). Neurochemically and Hodologically Distinct Ascending VGLUT3 versus Serotonin Subsystems Comprise the R2-*Pet1* Median Raphe. J. Neurosci. 41, 2581–2600. 10.1523/JNEUROSCI.1667-20.2021 33547164 PMC8018730

[B47] Silberberg G. Darvasi A. Pinkas-Kramarski R. Navon R. (2006). The Involvement ofErbB4 with Schizophrenia: Association and Expression Studies. Am. J. Med. Genet. 141B, 142–148. 10.1002/ajmg.b.30275 16402353

[B48] Skirzewski M. Karavanova I. Shamir A. Erben L. Garcia-Olivares J. Shin J. H. (2018). ErbB4 Signaling in Dopaminergic Axonal Projections Increases Extracellular Dopamine Levels and Regulates Spatial/working Memory Behaviors. Mol. Psychiatry 23, 2227–2237. 10.1038/mp.2017.132 28727685 PMC5775946

[B49] Teng T. Gaillard A. Muzerelle A. Gaspar P. (2017). EphrinA5 Signaling Is Required for the Distinctive Targeting of Raphe Serotonin Neurons in the Forebrain. eNeuro 4, 0327-16. 10.1523/ENEURO.0327-16.2017 PMC5292598 28197551

[B50] Ting A. K. Chen Y. Wen L. Yin D.-M. Shen C. Tao Y. (2011). Neuregulin 1 Promotes Excitatory Synapse Development and Function in GABAergic Interneurons. J. Neurosci. 31, 15–25. 10.1523/JNEUROSCI.2538-10.2011 21209185 PMC3078582

[B51] Walsh T. McClellan J. M. McCarthy S. E. Addington A. M. Pierce S. B. Cooper G. M. (2008). Rare Structural Variants Disrupt Multiple Genes in Neurodevelopmental Pathways in Schizophrenia. Science 320, 539–543. 10.1126/science.1155174 18369103

[B52] Wang H. Liu F. Chen W. Sun X. Cui W. Dong Z. (2018). Genetic Recovery of ErbB4 in Adulthood Partially Restores Brain Functions in Null Mice. Proc. Natl. Acad. Sci. USA 115, 13105–13110. 10.1073/pnas.1811287115 30498032 PMC6304932

[B53] Yamamuro K. Bicks L. K. Leventhal M. B. Kato D. Im S. Flanigan M. E. (2020). A Prefrontal-Paraventricular Thalamus Circuit Requires Juvenile Social Experience to Regulate Adult Sociability in Mice. Nat. Neurosci. 23, 1240–1252. 10.1038/s41593-020-0695-6 32868932 PMC7898783

[B54] Zhang D. Sliwkowski M. X. Mark M. Frantz G. Akita R. Sun Y. (1997). Neuregulin-3 (NRG3): a Novel Neural Tissue-Enriched Protein that Binds and Activates ErbB4. Proc. Natl. Acad. Sci. 94, 9562–9567. 10.1073/pnas.94.18.9562 9275162 PMC23218

[B55] Zhang S.-R. Wu J.-L. Chen H. Luo R. Chen W.-J. Tang L.-J. (2020). ErbB4 Knockdown in Serotonergic Neurons in the Dorsal Raphe Induces Anxiety-like Behaviors. Neuropsychopharmacol. 45, 1698–1706. 10.1038/s41386-020-0601-7 PMC7419508 31905370

[B56] Zhu X. Lai C. Thomas S. Burden S. J. (1995). Neuregulin Receptors, erbB3 and erbB4, Are Localized at Neuromuscular Synapses. EMBO J. 14, 5842–5848. 10.1002/j.1460-2075.1995.tb00272.x 8846777 PMC394702

