# Supplementary material for: Developmental Disruption of Erbb4 in Pet1+ Neurons Impairs Serotonergic Sub-System Connectivity and Memory Formation
Source: Front Cell Dev Biol. 2021 Dec 10;9:770458. doi: 10.3389/fcell.2021.770458 (PMC8703035; doi:10.3389/fcell.2021.770458)
Supplement: Supplementary file 1 [file DataSheet1.docx]

Supplementary Material

# Supplementary Data

## Supplementary Figures


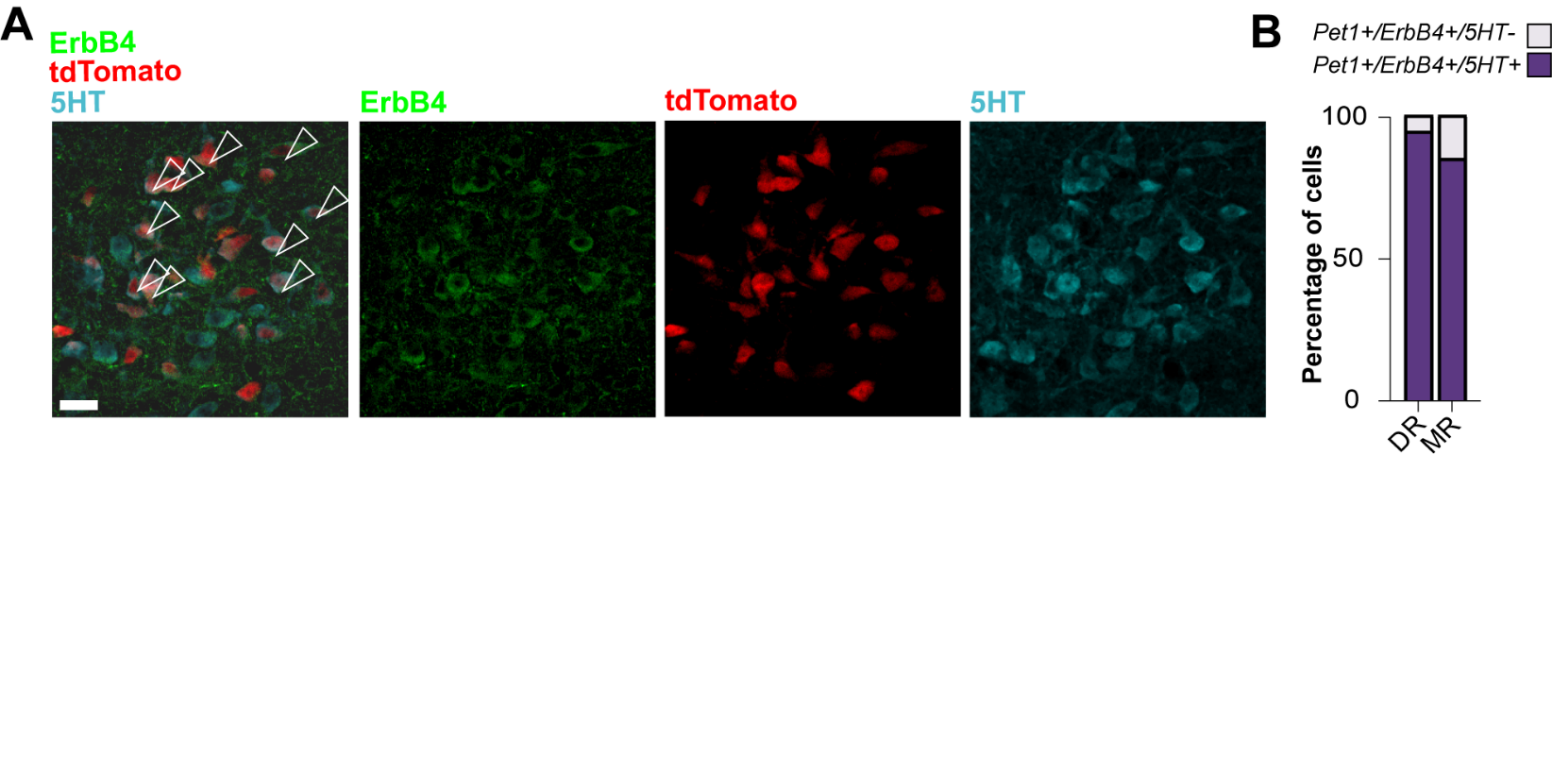


**Supplementary Figure 1. Colocalization of Pet1-tdTomato, ErbB4 and 5HT positive cells in DRN and MRN**. **(A)** Confocal images of Pet1-tdTomato (red), ErbB4 (green) and 5HT (cyan) in the dorsal raphe. Arrow heads point to triple positive cells for Pet1, ErbB4 and 5HT. **(B)** Quantification of the percentage of Pet1+ cells expressing Erbb4 and 5HT in the DR and MR nucleus (n = 5 mice). Scale bars for (A): 25µm.

**
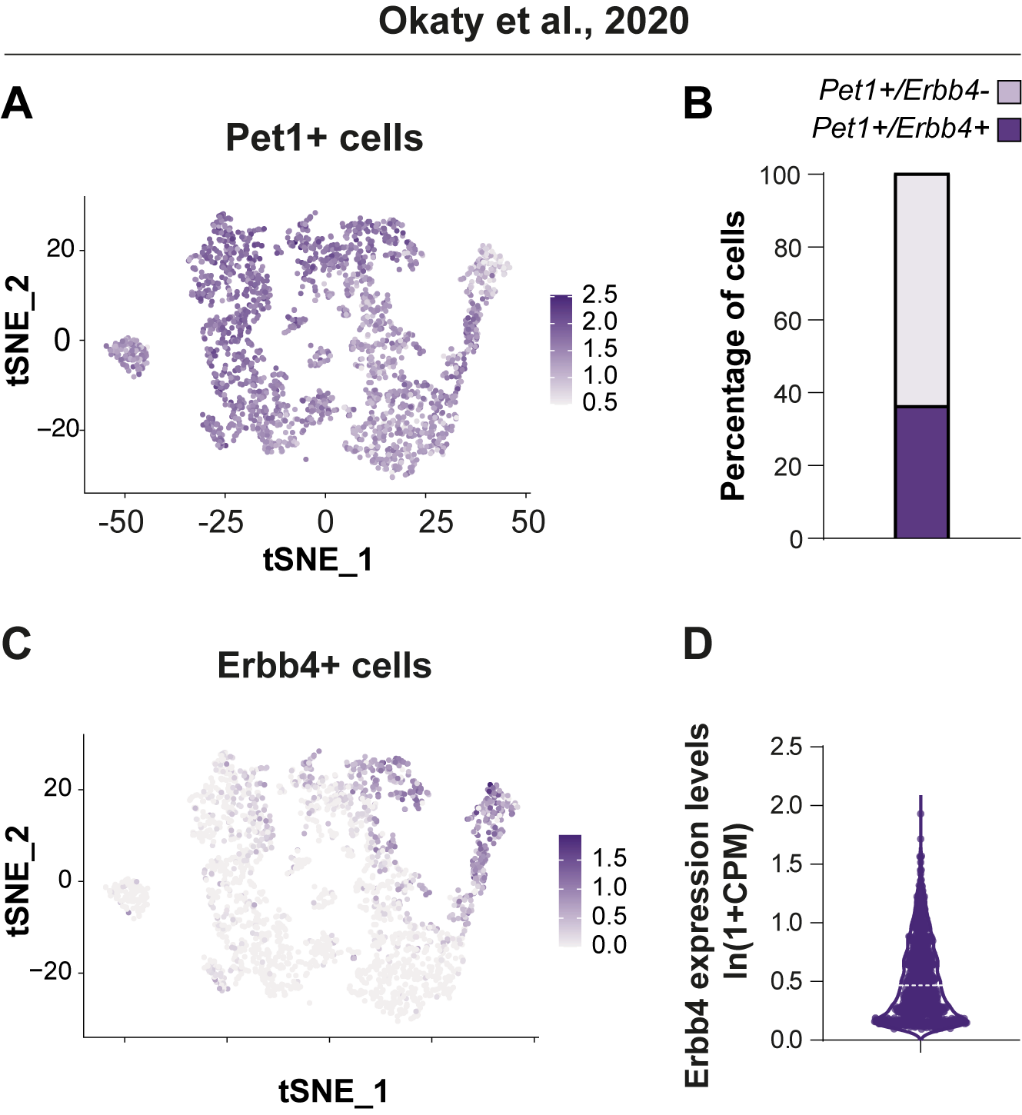
**

**Supplementary Figure 2. *Erbb4* transcripts displayed by adult Pet1+ neurons in Okaty et al., 2020 dataset.** **(A)** tSNE plot showing *Pet1* mRNA expression levels in the different clusters from the scRNA-seq dataset of Okaty et al., 2020. Cells are colored according to log-normalized expression levels of *Pet1* transcript. Color legend reflects expression values of *Pet1* ln (CPM+1). **(B)** Percentage of Pet1+ cells displaying *Erbb4* transcripts (37.32% of total Pet1 cells display *Erbb4* transcripts, i.e. 877 *Erbb4*+ cells from 2350 *Pet1*+ cells). **(C)** tSNE plot showing *Erbb4* mRNA levels in the different clusters from the original dataset. Cells are colored according to log-normalized levels of *Erbb4* transcript. Color legend reflects values of *Erbb4* transcripts in ln (CPM+1). **(D)** Violin plot representing the cell distribution according to *Erbb4* mRNA levels (ln (CPM+1)) within Pet1+/ErbB4+ cells (877 ErbB4+ cells display 0.49±0.01 reads (mean± SEM)).


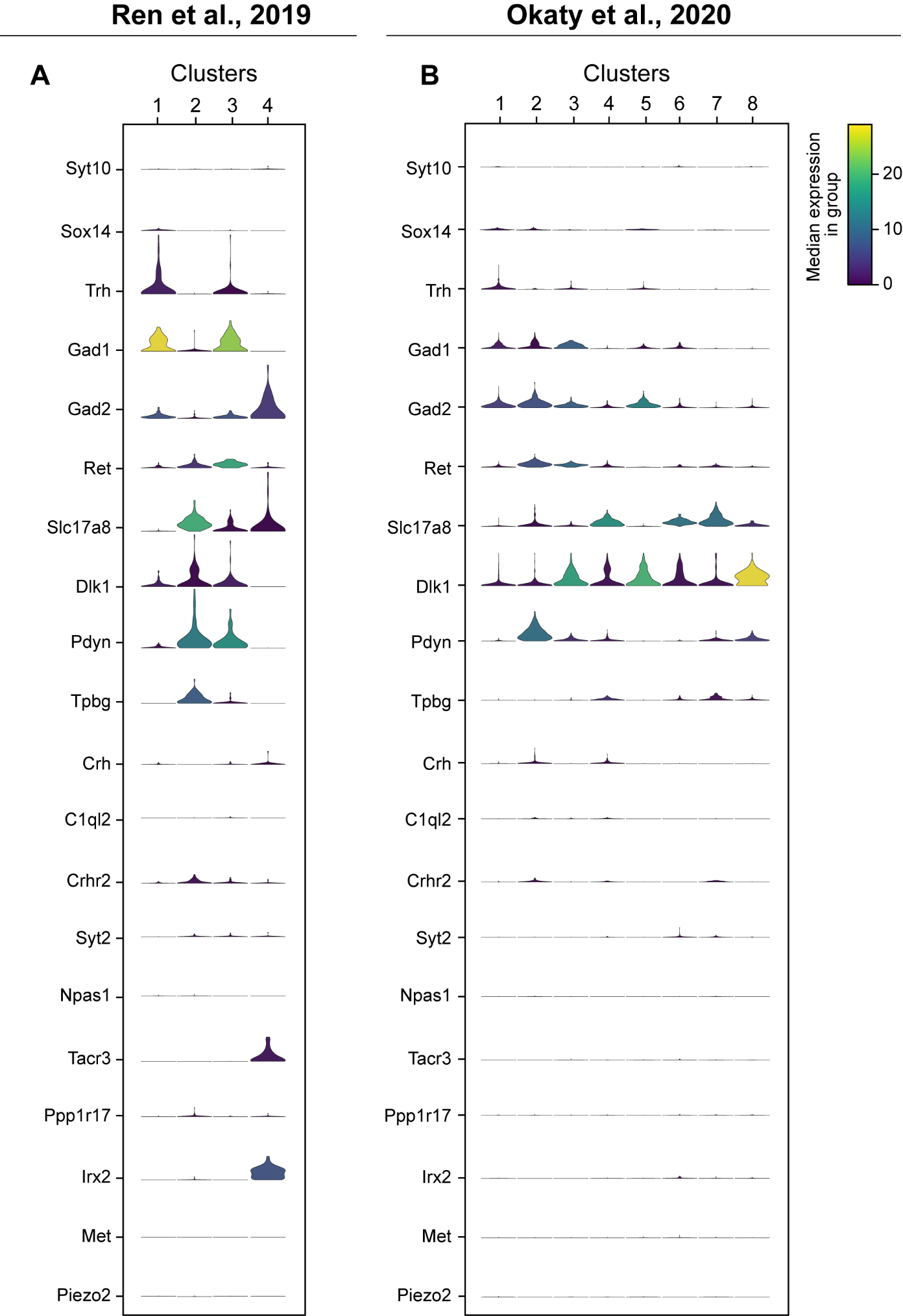


**Supplementary Figure 3. Violin plots of the expression of molecular marker genes for each *Erbb4+* cluster.** **(A)** Stacked violin plot representing the mRNA levels of different molecular markers for clusters that express *Erbb4* in the scRNAseq dataset from Ren et al., 2019. mRNA expression levels are displayed as ln(1+CPM). **(B)** Stacked violin plot representing the mRNA levels of different molecular markers for clusters that express *Erbb4* in the scRNAseq dataset from Okaty et al., 2020. mRNA levels are displayed as ln(1+CPM).


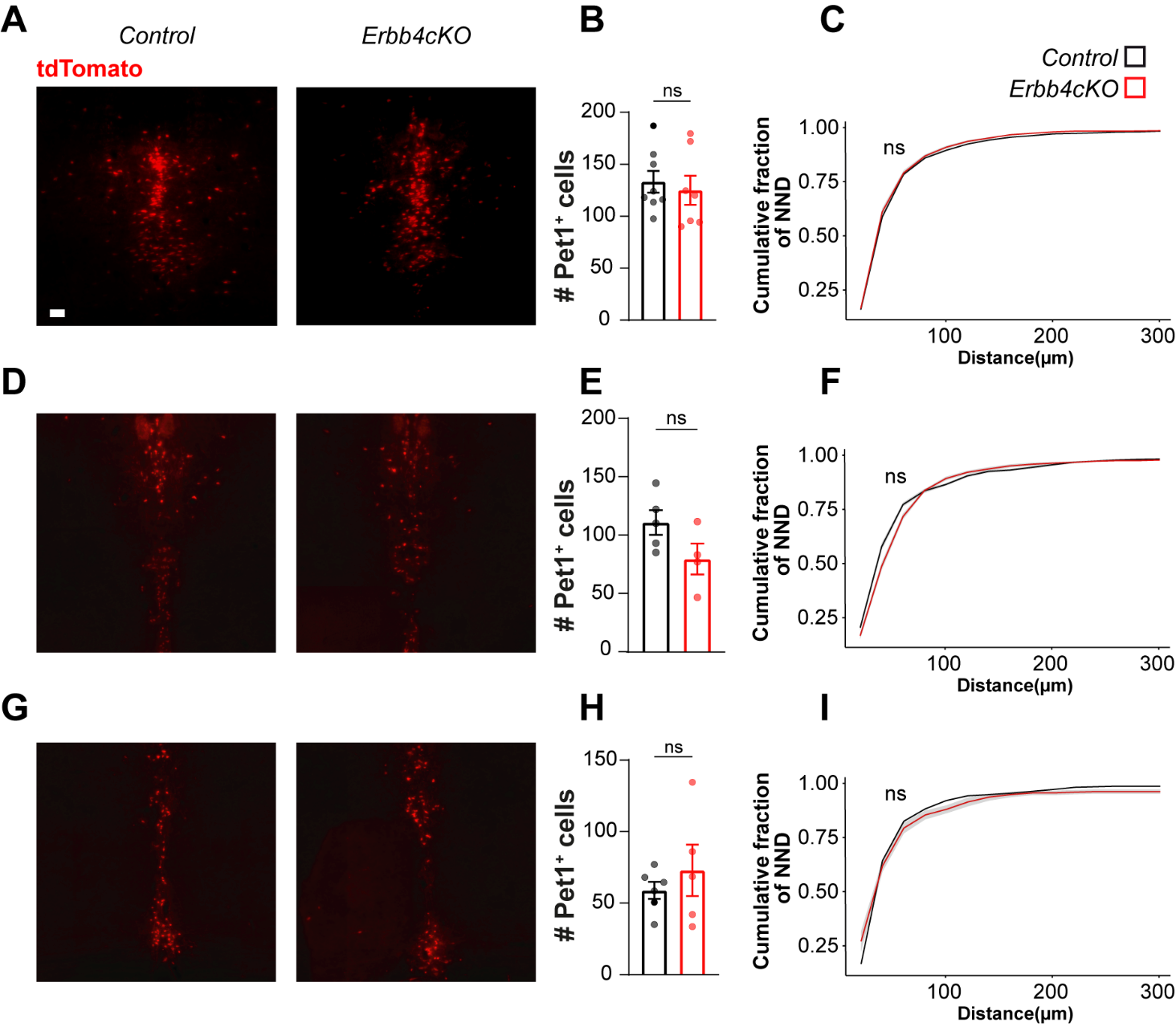


**Supplementary Figure 4. ErbB4 is not required for Pet1+ serotonergic neuronal migration in MRN.** (**A, D, G**) Distribution of Pet1-tdtomato+ neurons in the medial raphe nucleus (MRN). Coronal brain sections showing distribution of tdTomato+ neurons assessed at different levels of the MRN. **(B, E, H)** Quantification of total tdTomato+ neurons found at each coordinate as depicted in (**A, D, G**) in the MRN of control and *Erbb4cKO* mice shows non-significant differences (n = 5 –8 control and n = 4 – 7 mutant brains from 3 different litters). (**C, F, I**) Cumulative fraction of NNDs measured at each coordinate in the MRN shows non-significant differences in cell distribution (n = 5 –8 control and n = 4 – 7 mutant brains from 3 different litters). Scale bars in (**A, D, G**): 100µm. Data are represented as mean ± SEM. ns: not significant differences; t-test or Kolmogorov-Smirnov test.


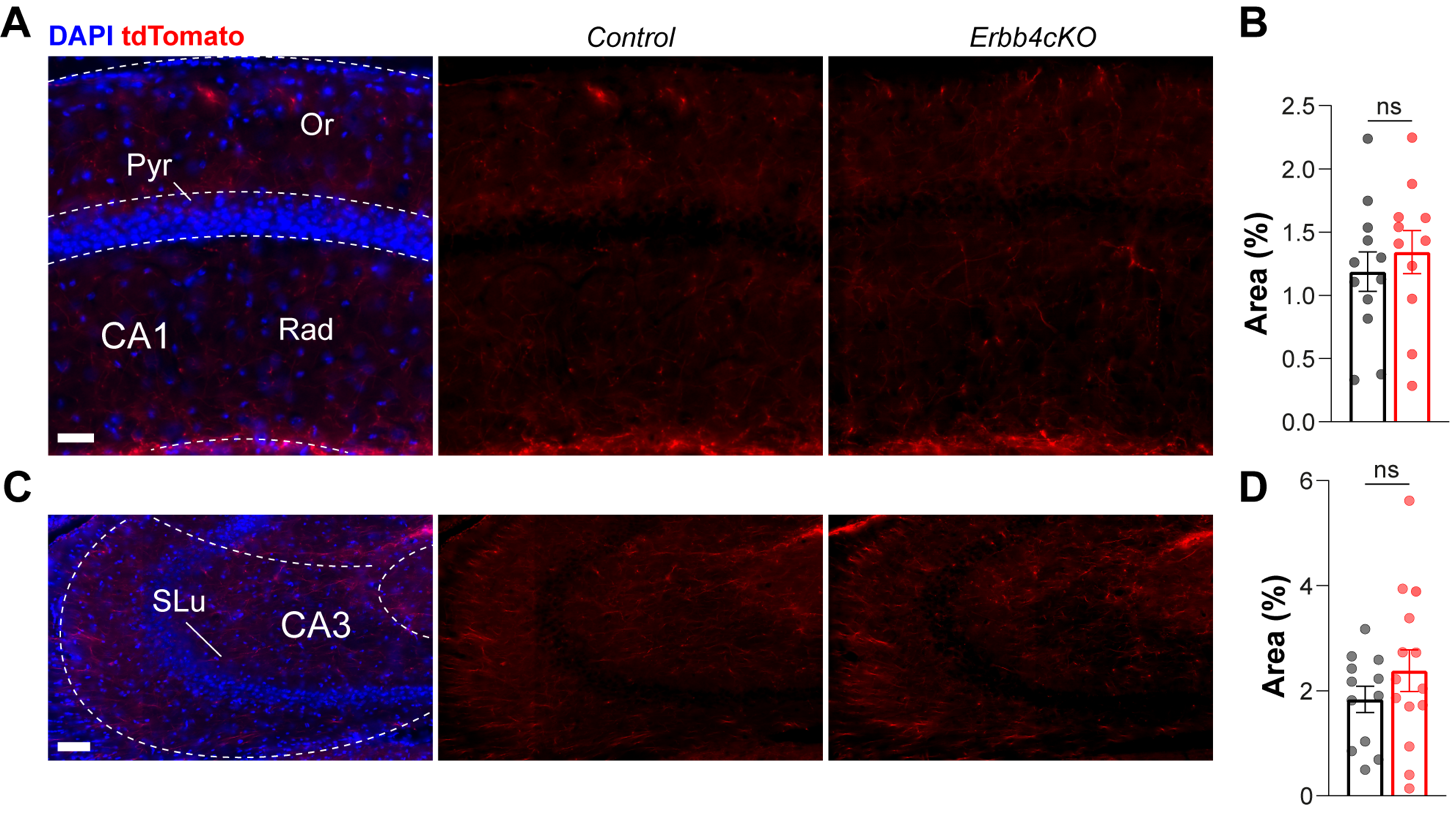


**Supplementary Figure 5. Long-range connectivity between Pet1+ circuits and CA1 and CA3 is not affected by *Erbb4*-deletion.** **(A, C)** Confocal images of Pet1+ tdTomato+ connectivity from raphe nucleus to CA1 and CA3 brain areas for control and *Erbb4cKO* mice. **(B, D)** Quantification of connectivity area normalized to the field of view are presented as percentage of area occupied by tdTomato signal (area%) (n=12 control and n=12-14 mutant from 3 different litters). Scale bars for (A): 50µm and (B): 100µm. Data are represented as mean ± SEM. ns: not significant differences; t-test or Kolmogorov-Smirnov test.


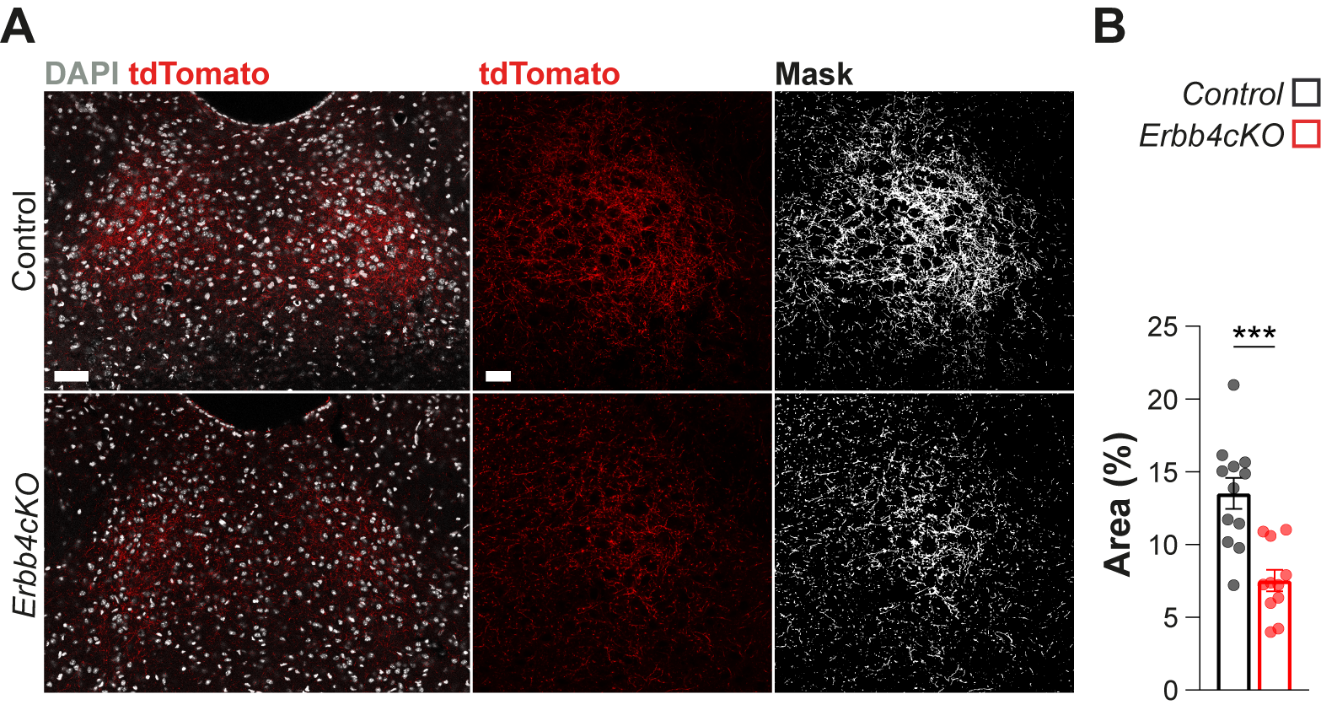


**Supplementary Figure 6. Long-range connectivity deficits in the periventricular thalamus (PVT) in mice with *Erbb4*-deletion in Pet1+ neurons.** **(A)** Confocal images of Pet1+tdTomato+ connectivity in the PVT region from control and *Erbb4cKO* mice. **(B)** Quantification of relative connectivity in PVT area presented as percentage of tdTomato signal normalized to total area (n=12 controls and n=11 mutants). Scale bar for **(A):** 50µm. Data are represented as mean ± SEM. ***: *p*<0.001*;* t-test.
